# Long-term effects of glyphosate on substrates with varying organic matter content: an ecotoxicological assessment under laboratory conditions

**DOI:** 10.1007/s11356-026-38047-6

**Published:** 2026-07-18

**Authors:** Olga Bemowska-Kałabun, Anna Zawadzka, Małgorzata Wierzbicka

**Affiliations:** 1 Department of Molecular Plant Physiology, Institute of Environmental Biology, Faculty of Biology, University of Warsaw, Ilji Miecznikowa 1, 02-096 Warsaw, Poland; 2 Department of Ecotoxicology, Institute of Environmental Biology, Faculty of Biology, University of Warsaw, Ilji Miecznikowa 1, 02-096 Warsaw, Poland

**Keywords:** Herbicide, Glyphosate, AMPA, Toxicity, Organic matter content of substrates, Biotests

## Abstract

The study aimed to determine the time required for glyphosate and its primary decomposition product, AMPA, to degrade in substrates with varying organic matter content, and to identify when the toxic effects of these substances diminish due to decomposition in the tested substrates. Additionally, the study investigated how nitrogen and phosphorus levels change during the decomposition of glyphosate and AMPA in relation to the organic matter content of the substrates. Ecotoxicological assessments were conducted using biotests (Phytotoxkit, *Lemna*, and Microtox). Chemical analyses of substrates with different organic matter contents were performed to measure glyphosate and AMPA levels, as well as various forms of nitrogen and phosphorus. The experiments were carried out under laboratory conditions over a time course from 0 to 24 months after herbicide application. It has been shown that sand is the substrate in which glyphosate and AMPA degrade most slowly (up to 24 months). The presence of undegraded glyphosate and AMPA in sand resulted in the longest-lasting toxic effects on test organisms in the bioassays conducted with this substrate. In agricultural soil, however, undecomposed glyphosate and AMPA had a toxic effect on the studied organisms only for a short period after the herbicide was applied, due to the rapid degradation of glyphosate and AMPA (3 months). There was an increase in the levels of phosphorus and nitrogen in the substrates after the herbicide was added, compared to the control. Although the experiments were conducted under laboratory conditions, the observed increase in nitrogen and phosphorus content in the tested substrates following the degradation of glyphosate and AMPA suggests that there may be potential implications for processes in the natural environment. This could be particularly relevant in areas where high doses of glyphosate have been used over many years. Additionally, it appears that in substrates with lower organic matter content, the degradation of glyphosate and AMPA might take longer, potentially leading to a more extended toxic effect on living organisms compared to substrates with higher organic matter. These observations could, in some cases, contribute to the development of increased tolerance to glyphosate in plants from such areas. However, further studies would be necessary to fully understand these effects.

**Supplementary Information:**

The online version contains supplementary material available at 10.1007/s11356-026-38047-6.

## Introduction

Glyphosate is a broad-spectrum, nonselective herbicide that inhibits the shikimic acid pathway involved in the biosynthesis of aromatic amino acids in plants and certain soil microorganisms. It is one of the most well-known and widely used active ingredients in herbicides. Glyphosate-based herbicides are applied globally in agriculture, along railway lines, for dam protection, in surface water systems, and in urban areas. The primary degradation product of glyphosate is AMPA (aminomethylphosphonic acid), which is also toxic to living organisms, although to a lesser extent than glyphosate itself (Baylis 2000; Gimsing et al. 2004; Peruzzo et al. 2008; Rampazzo 2009; Mensah et al. 2015; von Mérey et al. 2016; Padilla and Selim 2020; Singh et al. 2020a).

Glyphosate in the form of isopropylamine salt is contained in Roundup, a foliar, nonselective herbicide with systemic action (Baylis 2000; Rao 2014; Padilla and Selim 2020; Singh et al. 2020a, b). An interesting issue is the effect of glyphosate on poor soils, such as those found on railway embankments (crushed stone mixed with river sand). The most commonly used herbicides for controlling weeds on railway tracks are glyphosate-based agents, e.g., Roundup (Schweinsberg et al. 1999; Bӧrjesson and Torstensson 2000; Torstensson 2001; Torstensson et al. 2005; Burkhardt et al. 2008; Wierzbicka et al. 2015; Bemowska-Kałabun et al. 2021; Andersson et al. 2024; Buerge et al. 2024). The manufacturer’s recommendation for Roundup 360 SL (glyphosate in the form of isopropylamine salt—360 g per liter), when applied as a single treatment on railway tracks, is 7 L/ha (2378 g/ha glyphosate acid equivalent) or 3.5 L per km of track, covering a 5-m-wide weed zone. These are quite high quantities. For comparison, only 2–5 L/ha (679–1699 g/h glyphosate a.e.) are typically used to control weeds on fallow land (Roundup label 360 SL 2010). Additionally, a Roundup dose of less than 5 L/ha has been shown to be insufficient for effectively removing unwanted vegetation on railroad tracks (Bӧrjesson and Torstensson 2000; Torstensson 2001; Torstensson et al. 2005). For example, in Germany in 1990, the amount of herbicides used on railroad tracks was six times greater than that used in agriculture (Schweinsberg et al. 1999). This makes railway areas excellent sites for assessing the long-term effects of herbicide use. The application doses are high—even higher than in agricultural areas—and spraying occurs regularly (Bemowska-Kałabun et al. 2021).

Glyphosate is used on plants as a foliar agent; however, it also enters the substrate during spraying. According to the herbicide manufacturer and previous research, the half-life of glyphosate in soil ranges from 2 to 174 days (Schuette 1998; Safety data sheet Roundup 360 SL 2010; Safety data sheet Roundup Ultra 170 SL 2018), or even up to 215 days (Singh et al. 2020a). The degradation time for AMPA may be even longer—the half-life of AMPA is approximately 514.9 days (Singh et al. 2020a). An interesting environmental issue is the development of tolerance to glyphosate in plants. On the one hand, developing tolerance or even resistance to glyphosate is linked to its repeated use, especially in agricultural areas. Over time, microevolutionary processes lead to the emergence of biotypes that can withstand glyphosate in these treated regions. On the other hand, slower degradation of glyphosate and its metabolite AMPA, combined with prolonged exposure—particularly in soils with low organic matter content where high doses of glyphosate are applied—may also promote the development of plant tolerance. This situation is common in railway areas, where substrates typically has low organic matter. For example, a study by Bemowska-Kałabun et al. (2021) demonstrated increased glyphosate tolerance in *Geranium robertianum* L. growing in railway areas in northeastern Poland.

This paper is a continuation of the research conducted by Bemowska-Kałabun et al. (2021). It is an attempt to conduct an ecotoxicological analysis and to investigate, under controlled conditions, what happens to glyphosate and AMPA in substrates with different levels of organic matter after applying high doses of the herbicide, such as in railway areas. We were particularly interested in substrates with a low organic matter content, such as those found in railway areas. The objective of this study was (1) to determine the degradation rate of glyphosate in substrates with varying levels of organic matter; (2) to identify when glyphosate and its primary degradation product, AMPA, are no longer toxic to test organisms, and to relate these findings to the degradation rates of these substances in different substrates; and (3) to monitor changes in nitrogen and phosphorus levels over time during the degradation of glyphosate and AMPA in substrates with varying organic matter content. The experiments were conducted under controlled laboratory conditions. To evaluate the toxicity and degradation rates of glyphosate in substrates with different organic matter levels, both ecotoxicological tests using a series of bioassays and chemical analyses of the test substrates were performed. A comprehensive approach to the degradation of glyphosate and AMPA, their effects on living organisms, and the correlation of these factors with nitrogen and phosphorus levels over time has enabled an assessment of how these substances behave in substrates with varying organic matter content, thereby expanding our current understanding of the subject.

## Materials and methods

### Preparation of substrates for ecotoxicological and chemical analyses

The research material used in ecotoxicological studies and chemical analyses were substrates with varying organic matter content (Table 1):

- substrate with the highest content of organic matter: soil—the top 20 cm layer of soil collected from an agricultural area near Warsaw (Piastów), where no herbicides have been previously applied. The soils in Piastów are generally of good quality, dominated by lessivés and podzolic soils. Overall, Piastów’s soils are relatively undegraded and less disturbed compared to other regions in the Poland (Tomassi-Morawiec 2016; study-spatial development—state of the environment of the Piastów city 2025);
- substrate with medium organic matter content: sand mixed with soil in a 1:1 ratio;
- substrate with the lowest content of organic matter: only sand (P.P.H.U. “ZEW” company).

**Table 1:** The characteristics of the substrates (soil, sand with soil (1:1), and sand) utilized in the research

Parameter	Soil	Sand with soil (1:1)	Sand	Method	Scope of the method
pH^*^	7.82 ± 0.02	7.50 ± 0.02	7.33 ± 0.02	PN-ISO 10390:^1997^, PN-EN ISO 10390:^2022-09^ (potentiometric method)	3.0–10.0
Total organic carbon (% DM)^**^	15.1 ± 2.26	4.09 ± 0.61	< 0.10	CSN ISO 10694:^1995^, CSN EN 13137:^2002^, CSN EN 15936:^2022^	From 0.1
Humus content (%)^**^	26.0	7.05	< 0.1724	Calculation method: Humus content (%) = C_org_ (equal to TOC) × 1.724 (e.g., Costea, Tăuşan 2025)	From 0.1724
Salinity (%)^**^	0.20 ± 0.01	< 0.10	< 0.10	PN-ISO 11265:^1997^, PN-EN ISO 11265:^2026-03^	0.10–99

^*^The pH levels were tested at the accredited laboratory of Hamilton UO-Technologia Sp. z o.o. (J.S. Hamilton Poland)

^**^The measurements of total organic carbon, humus content, and salinity were performed at the accredited research laboratory INTERLABO

In this study, the same dose of Roundup herbicide used on railway tracks over five years was applied. The calculations assumed double spraying per year with the manufacturer-recommended dose (Roundup label 360 SL 2010; Safety data sheet Roundup 360 SL 2010). When determining the dose, 100% spray efficiency was assumed, without considering additional variables. Based on these calculations, a 25 mL solution containing 7200 mg/L of glyphosate (which equals 180,000 µg of glyphosate) was used for every 0.5 kg of substrate (equivalent to 360 mg/kg of glyphosate in each substrate). The herbicide solutions were prepared using a measuring cylinder and then thoroughly mixed with the substrates to ensure even distribution. For preparing the solutions, Roundup Ultra 170 SL was used, which contains 170 g of glyphosate per liter (Safety data sheet Roundup Ultra 170 SL 2018). The prepared substrate was stored in pots at room temperature (23 ± 2 °C during the day and 18 ± 2 °C at night) and watered regularly—twice a week with 100–250 mL of water, depending on the drying level of the substrate—to keep it moist and prevent it from drying out or water from flowing out of the saucers under the pots. A negative control was always included, consisting of substrates (soil, soil mixed with sand, or sand only) without any glyphosate. Control pots were maintained under the same conditions as the test samples. Measurements, including biotests and chemical analyses, were conducted on the control samples simultaneously with those of the test samples.

### Phytotoxkit biotest

The Phytotoxkit plant biotest is a short-term, chronic test that evaluates root growth. It involves counting the number of germinated seeds and measuring the length of their roots after several days of exposure to toxic substances in the medium, compared to a control medium. The Phytotoxkit procedure follows the standards outlined in ISO 11269-1 (Phytotoxkit, Phytotoxkit. Standard operational procedure). This method for assessing root growth inhibition is useful for testing the toxicity of chemical compounds, except for volatile substances or those that primarily affect photosynthesis (ISO 11269-1:^2012^; PN-EN ISO 11269-1:^2013-06^; Wierzbicka et al. 2015).

Phytotoxkit biotests were conducted at the following time points: 1 day, 1 month, 3 months, 6 months, 9 months, 13 months, and 24 months after herbicide application to the substrate. Substrate samples were collected at each of these intervals and placed into Phytotoxkit plates (90 cm^3^ each). The substrates were then soaked in distilled water according to their specific water holding capacity. Each plate was covered with filter paper, and 10 seeds of the test plant were evenly spaced in a line on the filter. The tests were performed following the standard biotest procedure (Phytotoxkit, Phytotoxkit. Standard operational procedure; ISO 11269–1:^2012^; PN-EN ISO 11269-1:^2013-06^; Wierzbicka et al. 2015). The test plants were incubated with the substrates in the dark at 25 °C for seven days. The test plates were scanned and photographed with a computer scanner every 24 h during the first four days and also on the seventh day (72 h after the previous measurement) to monitor changes over a longer period. Root length was measured with the Image Tool software (UTHSCSA ImageTool 3.0 2012). These measurements allowed us to calculate the root toxicity index (IT) using the formula: IT = [(*A* – *B*)/*A*] × 100, where A is the root length in the control substrate, and B is the root length in the test substrate (Phytotoxkit. Standard operational procedure). Each test was performed in five replicates (five test plates with 10 seeds each, totaling 50 seeds per tested medium). The Phytotoxkit biotests used *Lepidium sativum* L. (garden cress), as recommended by the manufacturer. This species is commonly used in ecotoxicological studies (Czerniawska-Kusza et al. 2006; Phytotoxkit, Phytotoxkit. Standard operational procedure; Wierzbicka et al. 2015). Control samples consisted of substrates without herbicide addition. Statistical analyses were performed.

### *Lemna* test

The *Lemna* test (duckweed test) is used to evaluate the toxicity of a specific environmental sample. The test involves the freshwater plant *Lemna minor* L., commonly known as duckweed. Due to its small size, simple structure, ability to reproduce vegetatively, and rapid division, this plant is frequently used in research related to water quality, municipal and industrial wastewater, and soil extracts (Brain et al. 2004; ISO 20079:^2005^; Kuczyńska et al. 2005; Robinson et al. 2005; OECD 2006; PN-EN ISO 20079:^2006^; Bielińska and Nałęcz-Jawecki 2009; Mkandawire et al. 2014; Drobniewska et al. 2017).

The *Lemna* test was conducted 1 day and 14 months after the herbicide was applied to the substrate. Water extracts of the tested soil substrates were examined (with and without herbicide). The test was performed according to OECD standards (OECD 2006). Before testing, *L. minor* was cultured in the Swedish Standard (SIS) medium, without the optional MOPS buffer, which is recommended for this species and aligns with OECD guidelines (2006). The cultures were maintained under a consistent photoperiod: daytime at 23 °C with 16 h of light at 10,000 lx, and nighttime at 20 °C for 8 h. The test included six replicates. Control plants were grown in the same Swedish Standard (SIS) medium. The reference trials, compared to the herbicide treatments, involved water extracts from individual substrates without the addition of herbicide (blank application), aside from the control (Swedish Standard medium). The test was conducted in polystyrene microplates (6 cells in a container) with a lid. Each cell contained a single plant consisting of 3–4 fronds. For preparing extracts of the tested substrates, 10 g of substrate samples were weighed out, placed in sterile plastic containers, and poured over with 50 mL of distilled water. The prepared solutions were mixed in a vortex mixer for 30 min and then left at room temperature for 24 h. Next, each sample was shaken for another 30 min. After the pellets had settled, 8 mL of the water extract and 1000 µL of the pellet were transferred to each test cell. The temperature in each test cell was measured every other day and ranged between 24 ± 2 °C, meeting the standard criteria. The reaction of the tested media, incubation temperature, and light intensity also complied with the standard requirements. The number of fronds and the overall condition of the plants were assessed daily for 7 days—on the day of setting up the test and after 6 days of incubation (OECD 2006; Bielińska and Nałęcz-Jawecki 2009; Drobiewska et al. 2017).

The plants were observed using a binocular microscope equipped with a digital camera and an image analysis system (NIS-Elements BR 3.0, Nikon). Photos were also taken daily with the camera. After the experiment was completed, the fresh and dry weights of the plants from each treatment were measured. To determine dry weights, the plants were dried in a laboratory oven at 50 °C for 24 h until a constant weight was reached. Data collected included the numbers of plants and fronds, and the dry and fresh weights of the plants. Additionally, the frond area for each experimental variant was measured using ImageJ software (Ferreira and Rasband 2012).

The mean and standard deviation of the increase in the number of fronds and plant area on consecutive days during the test were calculated. The average specific growth rate was determined for both plant area and number of fronds using the formula *R* = (ln *x*_*t*2_ – ln *x*_*t*1_)/(*t*_2_ – *t*_1_), where *R* is the specific growth rate per day, *x*_*t*1_ is the value of the test response at time *t*_1_ (the day the test was initiated), *x*_*t*2_ is the value at time *t*_2_ (the test end date), and *t*_2_ – *t*_1_ is the time interval between measurements in days. The growth inhibition factor was also calculated for plant area and number of fronds using IR = [(*R*_*c*_ – *R*_*t*_)/*R*_*c*_] × 100, where IR is the growth inhibition factor, *R*_*c*_ is the growth in the control, and *R*_*t*_ is the growth in the test sample. The percentages of water content and dry weight were calculated as follows: H_₂_O [%] = [(*A* – *B*)/*A*] × 100, and dry matter [%] = (*B*/*A*) × 100, where *A* is the fresh weight and *B* is the dry weight (OECD 2006; Bielińska and Nałęcz-Jawecki 2009; Drobiewska et al. 2017). Statistical analyses were performed.

### Microtox biotest

Microtox is a highly sensitive test for acute toxicity that uses the marine bacteria *Vibrio fischeri* as the test organisms. The assay measures how substances inhibit the bacteria’s bioluminescence (Kuczyńska et al. 2005; Ghirardini et al. 2009; Microtox® M500. Industry-leading toxicity detection; Wierzbicka et al. 2015).

The test was conducted on substrates with the herbicide applied at 1 day, 16 months, and 24 months after treatment. The control sample consisted of a 2% NaCl solution, as recommended by the manufacturer. In addition to the control, extracts from substrates that did not contain the herbicide were included as reference samples. The Microtox Basic Solid Test (screening test) was used to evaluate the solid samples. Before testing, 100% extracts of the substrates were prepared. For this procedure, a 7 g sample of the substrate was mixed with 35 mL of diluent solution (2% NaCl solution) in a sterile plastic container. The prepared solutions were shaken for 30 min. According to the manufacturer’s recommendations, the optimal pH range for the samples is between 6.5 and 8.5, within which *V. fischeri* exhibits optimal luminescence. Any deviations from this pH range should be corrected using 1 M NaOH or 1 M HCl. In the present study, the pH of the samples remained within the specified range. A bacterial suspension was prepared before conducting the tests. The reconstructive solution was added to the bacteria in a freeze-dried form. The test was conducted following the standard protocol. Tubes containing the test solutions were placed in an incubator set to 15 °C and connected to the Microtox M500 photometer for measurement. A 200 μL suspension of reconstructed bacteria was added to each test tube. Calibration of the photometer was performed in accordance with the test procedure. Measurements were taken at 1, 5, 15, and 30 min after the start of the test. Results were expressed as the percentage of light inhibition relative to the control (that is, 2% NaCl solution) at each time point. The assay was performed in duplicate according to the standard protocol (Ghirardini et al. 2009; Wierzbicka et al. 2015; Microtox Microtox® M500. Industry-leading toxicity detection). Statistical analyses were carried out on the data.

### Chemical analyses of substrates treated with herbicide

The decomposition rate of Roundup herbicide in substrates with different levels of organic matter content was studied. The contents of glyphosate and its primary degradation product, AMPA, were measured after 5 days and at 1, 6, 9, 12, and 24 months following herbicide application. Additionally, after 5 days and 6, 12, and 24 months, the levels of soluble phosphorus, total phosphorus, total nitrogen, ammonium nitrogen, nitrate nitrogen, and soluble forms of nitrogen in the media were also analyzed. Since individual mixed samples were tested for each variant, no statistical analyses were performed. The graphs from the chemical analyses display results with expanded uncertainty at a 95% confidence level, using a coefficient *K* = 2.

The glyphosate and AMPA content tests were conducted at the accredited laboratory of Hamilton UO-Technologia Sp. z o.o. (J.S. Hamilton Poland), while the phosphorus and nitrogen content tests were performed at the accredited laboratory of ALS Czech Republic s.r.o. Each time, 1 kg of substrate was submitted for chemical analysis. Glyphosate and AMPA concentrations were determined using the LC/MS/MS method, following the protocol established by Anastassiades et al. (2019). This methodology was also employed in our previous studies (Bemowska-Kałabun et al. 2021).

The determination of nitrate nitrogen, ammonium nitrogen, and total soluble nitrogen was carried out in accordance with ISO 14255:^1998^ (determination of nitrate nitrogen, ammonium nitrogen, and total soluble nitrogen in air-dried soils using calcium chloride as an extractant by the CFA method). Total nitrogen determinations were performed according to ISO 11261:^1995^ (modified Kjeldahl method). Soluble phosphorus determinations were conducted following ISO 11263:^1994^ (spectrophotometric determination of phosphorus soluble in sodium bicarbonate solution). Total phosphorus was determined according to EPA Method 3050B U.S. EPA (1996), which involves element detection by atomic emission spectrometry.

### Statistical analysis

Statistical analyses of the biotest results were performed using STATISTICA software, version 13.1 (StatSoft, Inc. 2014). The Shapiro–Wilk test was employed to assess the normality of data distribution, while Levene’s test checked for homogeneity of variances. Both tests were conducted for each dataset. Depending on the results, either ANOVA followed by a post hoc Tukey test or a nonparametric Kruskal–Wallis ANOVA test was used, followed by a multiple comparison of mean ranks for all samples (Kruskal–Wallis test for multiple independent samples). All analyses were performed at a significance level of *α* < 0.05. Asterisks (*) denote statistically significant differences between herbicide-treated substrates and the corresponding control. Bars without any shared letters are considered significantly different. The charts illustrating the biotest results display arithmetic means accompanied by standard deviations. In addition, the results of the most important statistical analyses, including *p*-values, are presented in tabular form in an Excel file included in the supplementary materials.

## Results

### Substrate with the lowest organic matter content—sand

#### Phytotoxkit

**Fig. 1:**
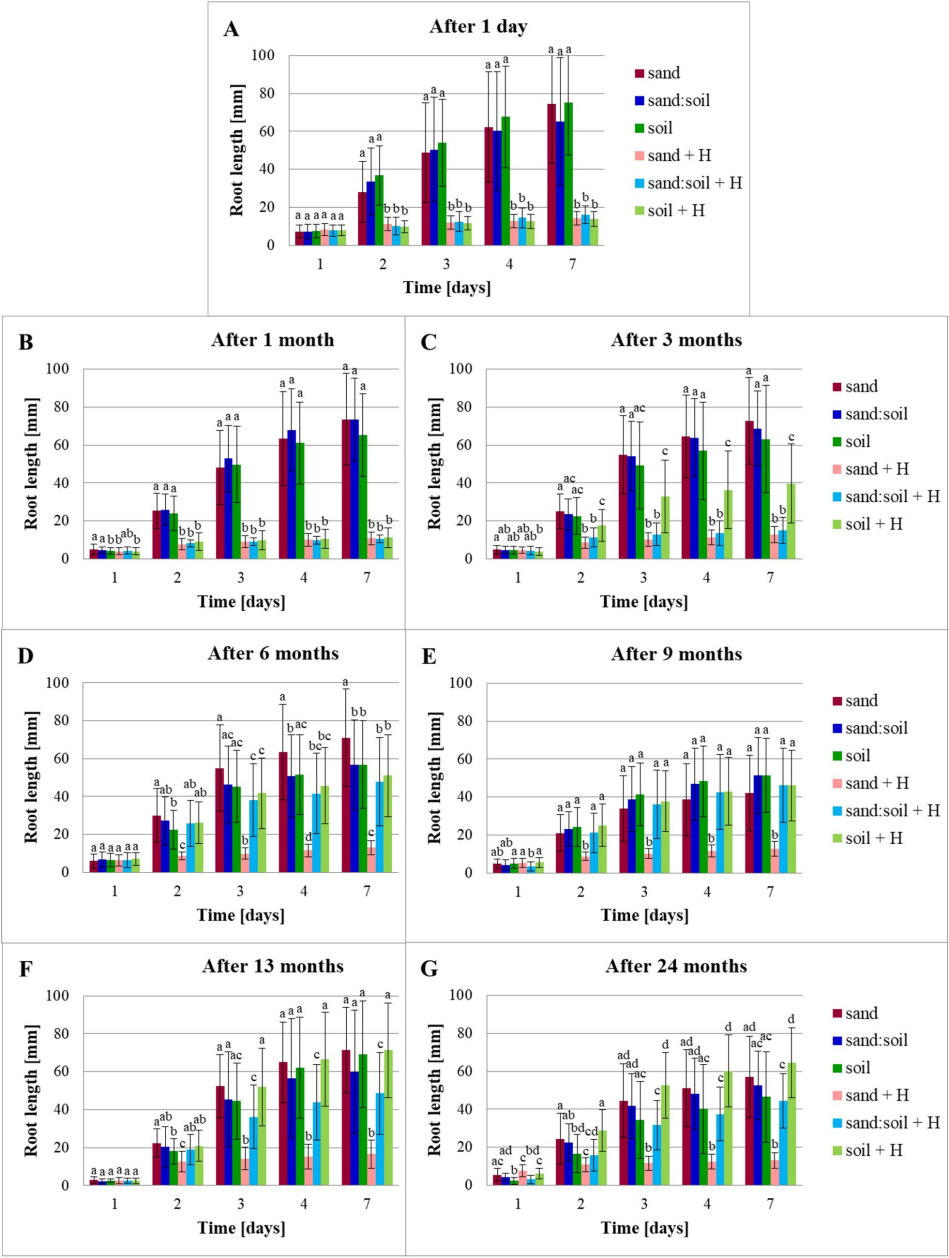
Root length of *L. sativum* grown on tested and control substrates (sand, sand with soil, and soil). The measurements were taken at various times after herbicide application: 1 day (**A**), 1 month (**B**), 3 months (**C**), 6 months (**D**), 9 months (**E**), 13 months (**F**), and 24 months (**G**). Abbreviations: + H—samples treated with herbicide. The charts display mean values with standard deviations. Bars that do not share any letters are significantly different, as determined by the Kruskal–Wallis ANOVA followed by a multiple comparison of mean ranks (Kruskal–Wallis test for multiple independent samples); significance level *α* = 0.05. In addition, the results of the statistical analysis (Kruskal–Wallis test for multiple independent samples) are presented in Table S1—a supplementary Excel file

**Fig. 2:**
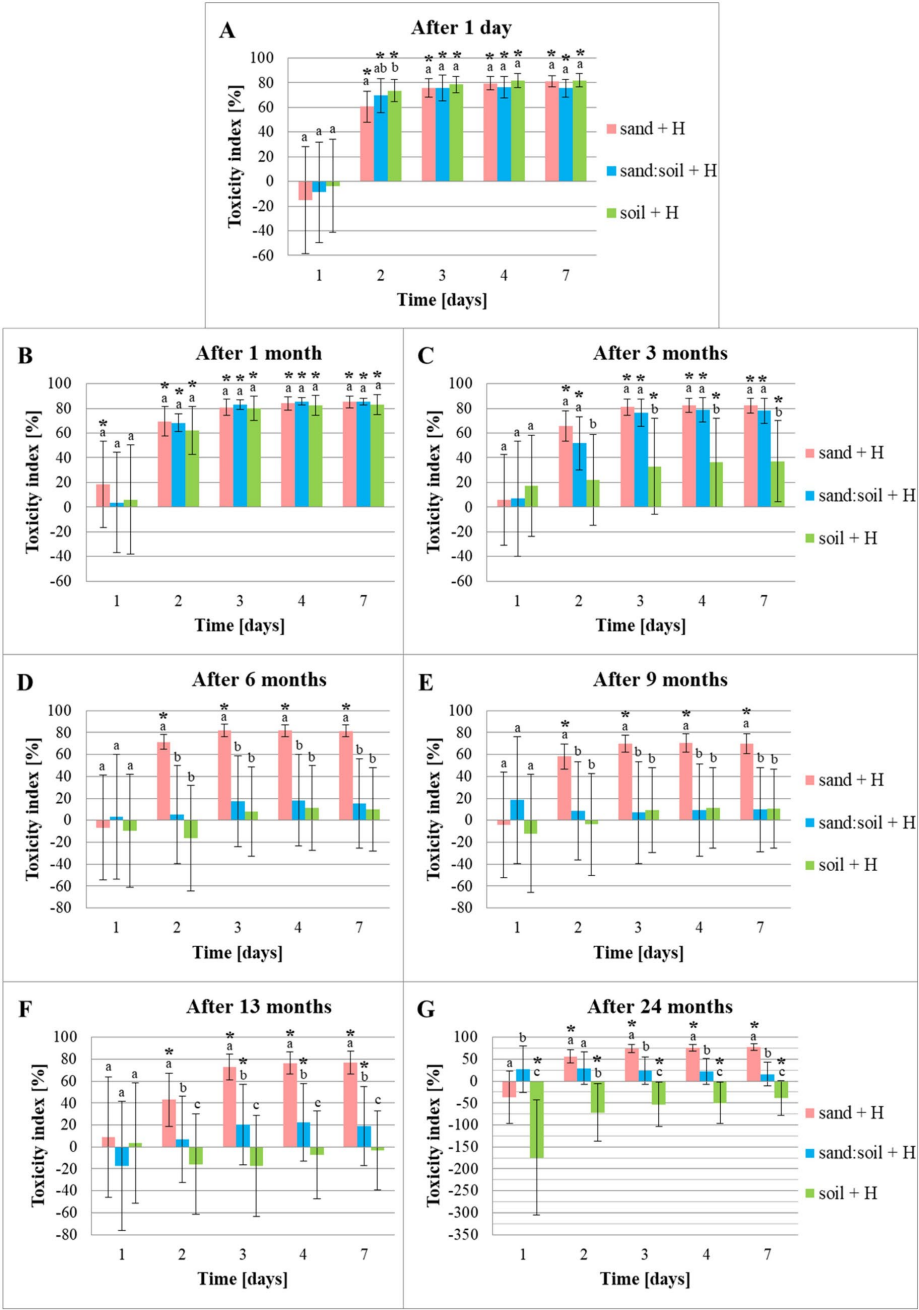
Root toxicity index of *L. sativum* grown on tested and control substrates (sand, sand with soil, and soil). A higher toxicity index indicates greater toxicity of the substrate. The measurements were taken at various times after herbicide application: 1 day (**A**), 1 month (**B**), 3 months (**C**), 6 months (**D**), 9 months (**E**), 13 months (**F**), and 24 months (**G**). Abbreviations: + H—samples treated with herbicide. The charts display mean values with standard deviations. Asterisks (*) indicate statistically significant differences between the tested herbicide-treated substrates and the control. Bars that do not share any letters are significantly different, as determined by the Kruskal–Wallis ANOVA followed by a multiple comparison of mean ranks (Kruskal–Wallis test for multiple independent samples); significance level *α* = 0.05. In addition, the results of the statistical analysis (Kruskal–Wallis test for multiple independent samples) are presented in Table S2—a supplementary Excel file

In the variant where the herbicide was added to the sand, the lowest growth of *L. sativum* roots was observed—less than 20 mm. This result remained consistent at all time points, even after 24 months (Fig. 1; Supplementary materials, Table S1). The root toxicity index for plants incubated in sand with glyphosate was approximately 80% across all time points (Fig. 2; Supplementary materials, Table S2). The differences in root length between the herbicide-treated variant and the control were statistically significant at each of the tested time points, typically between days 2 and 7 of the biotest (Figs. 1 and 2; Supplementary materials, Table S1 and S2). In the herbicide-treated variants, statistically significant differences in the toxicity index between sand (sand + H) and soil (soil + H) appeared after 3 months. Similarly, differences between the sand with soil (sand:soil + H) and the sand-only variant (sand + H) emerged after 6 months. In both cases, these differences persisted up to 24 months (Fig. 2; Supplementary materials, Table S2).

The root length of the control plants grown in the substrate with the lowest organic matter content—sand—was approximately 40 to 70 mm at all time points (1 day; 1, 3, 6, 9, 13, and 24 months). The differences in root length between *L. sativum* plants grown on sand without herbicide and those on other control media were not statistically significant (Fig. 1; Supplementary materials, Table S1).

#### *Lemna* test

**Fig. 3:**
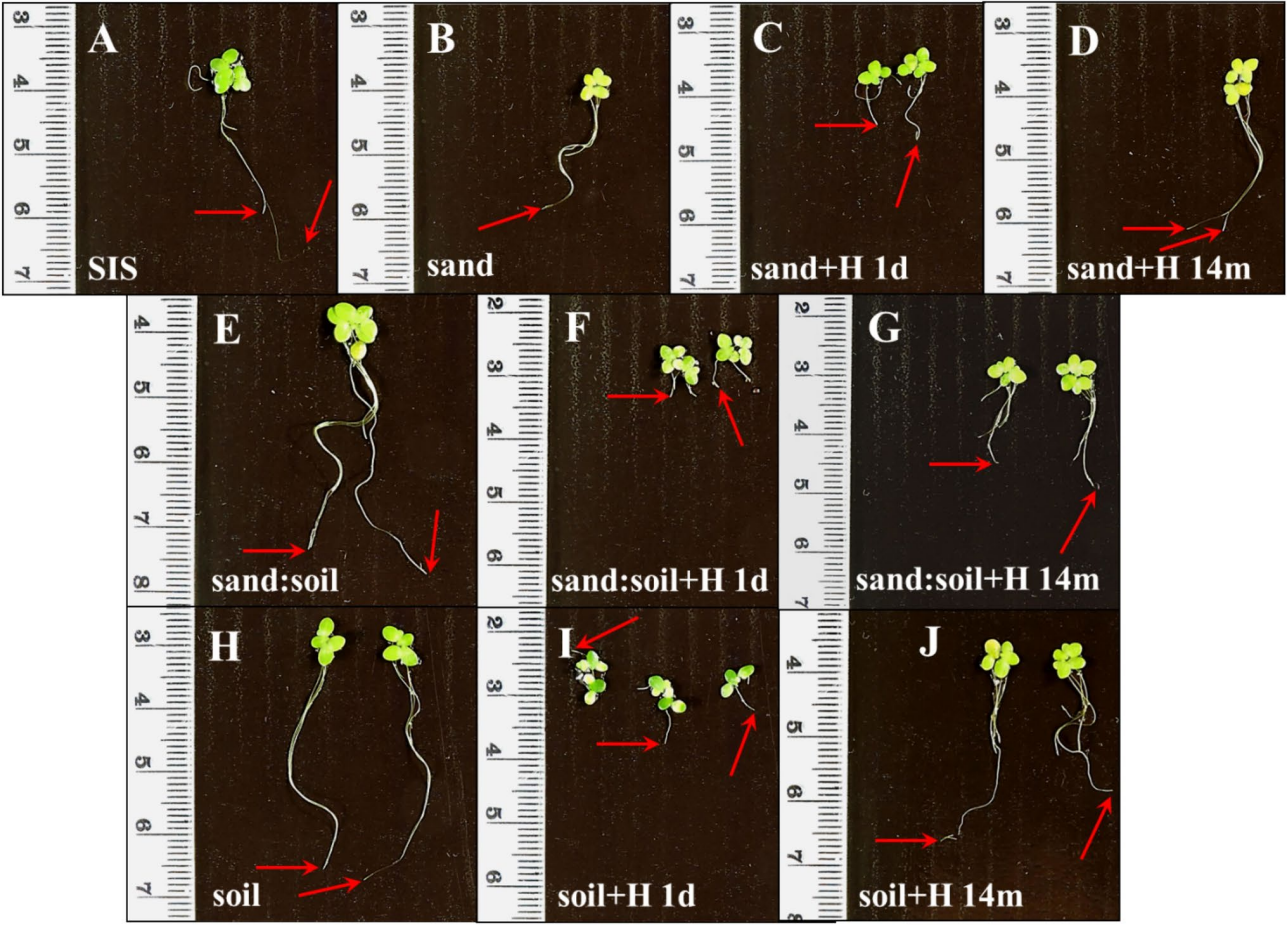
*L. minor* from the tested variants on the last day of the experiment. **A** Swedish Standard (SIS) medium (control), **B–D** sand, **E–G** sand with soil, and **H–J** soil. Abbreviations: + H—samples with herbicide (**C**, **D**, **F**, **G**, **I**, **J**); 1d—one day (**C**, **F**, **I**), 14 m—fourteen months (**D**, **G**, **J**) after herbicide application. The photos were taken with a camera. Red arrows indicate the tips of the roots of *L. minor*

**Fig. 4:**
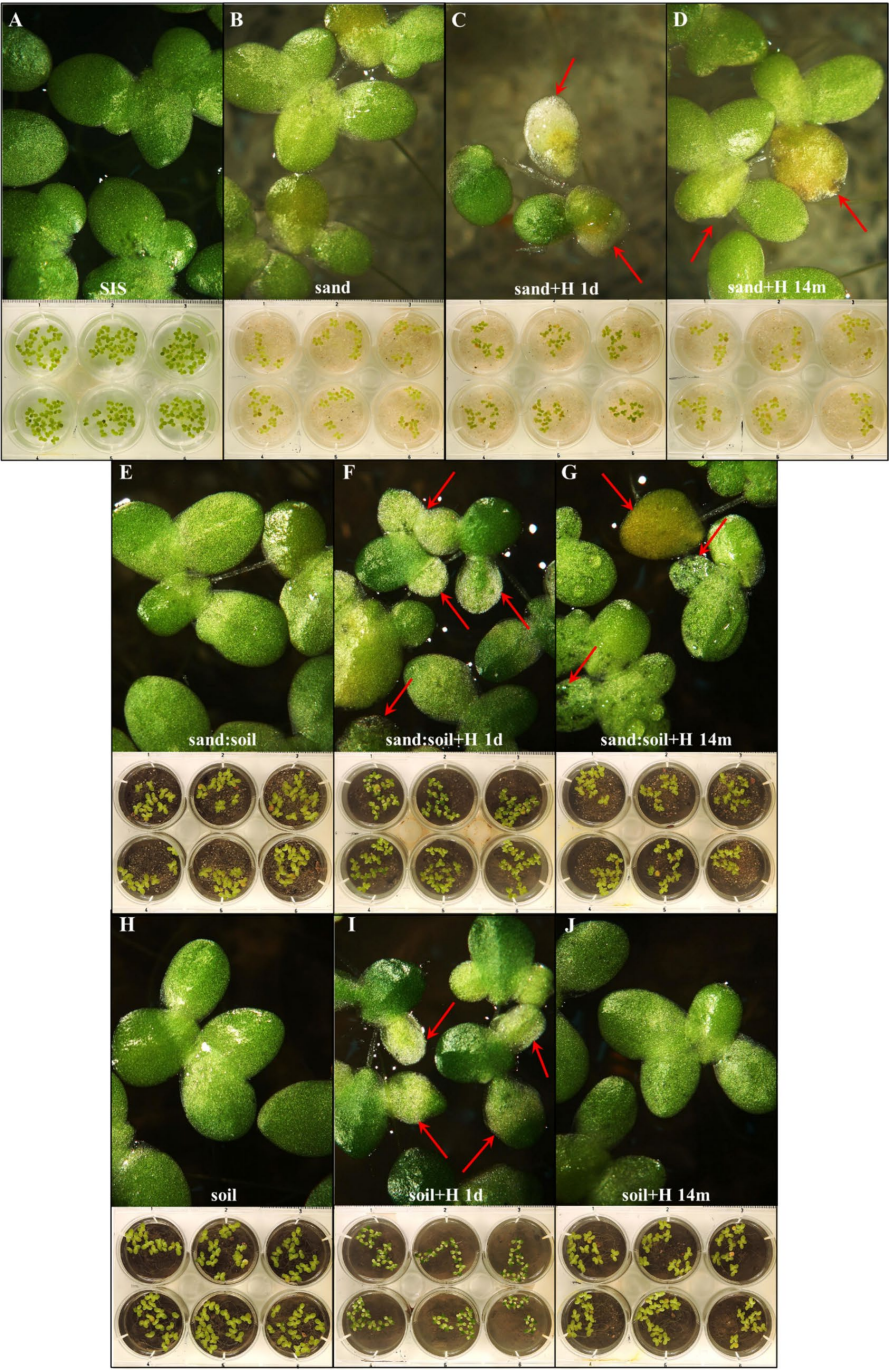
*L. minor* from the tested variants on the last day of the experiment. **A** Swedish Standard (SIS) medium (control), **B** sand, **E** sand with soil, and **H** soil. Abbreviations: + H—samples with herbicide (**C**, **D**, **F**, **G**, **I**, **J**); 1d—one day (**C**, **F**, **I**); 14 m—fourteen months (**D**, **G**, **J**) after herbicide application. Photographs of the plants were taken using a binocular microscope (magnification 1.5 ×); images of the entire test plates were captured with a camera. Red arrows indicate changes in the *L. minor* fronds (chlorosis and necrosis)

**Fig. 5:**
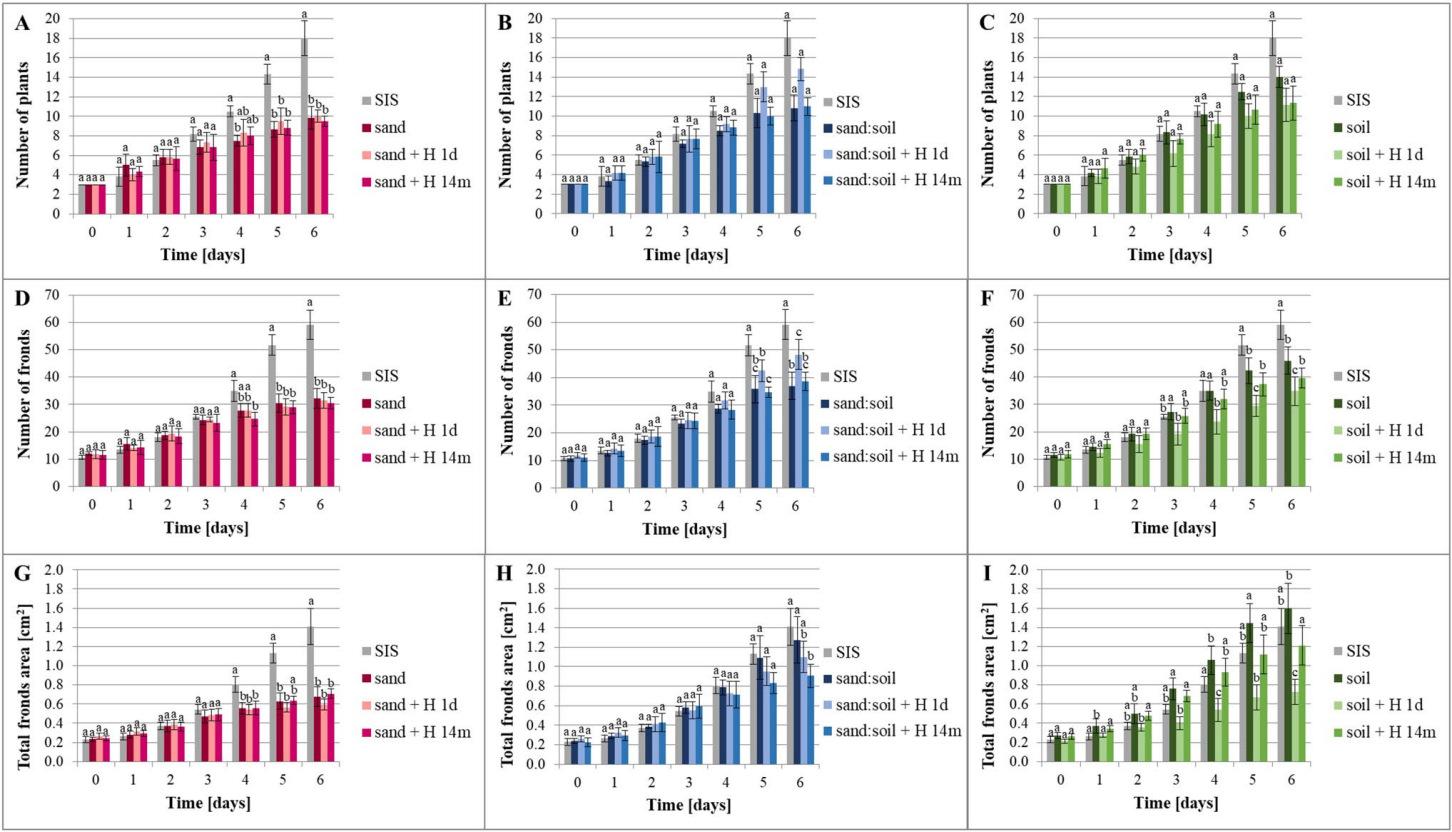
*Lemna* test results: number of plants (**A**–**C**), number of fronds (**D**–**F**), total frond area (cm^2^) (**G**–**I**) during the experiment. The figures represent the Swedish Standard (SIS) medium (control), as well as variants with water extracts of sand (**A**, **D**, **G**), sand mixed with soil (**B**, **E**, **H**), and soil alone (**C**, **F**, **I**). Each was tested both without and with herbicide. Abbreviations: + H—samples treated with herbicide; 1d—one day after application; 14 m—fourteen months after herbicide application. The charts display arithmetic means with standard deviations. Bars that do not share any letters are significantly different, as determined by the Kruskal–Wallis ANOVA followed by a multiple comparison of mean ranks (Kruskal–Wallis test for multiple independent samples); significance level *α* = 0.05. The average specific growth rate (*R*) and the percentage inhibition of growth rate (IR) for the parameters studied are presented in Table 2. In addition, the results of the statistical analysis (Kruskal–Wallis test for multiple independent samples) are presented in Table S3—a supplementary Excel file

The growth of *L. minor* plants in the sand variants was weaker compared to the control in the variant without herbicide, as well as in both sand variants with added herbicide (Figs. 3 and 5, Table 2; Supplementary materials, Table S3). One day after the herbicide application, the plants exhibited strongly shortened roots (~ 1 cm) and chlorosis. In contrast, these symptoms were not observed in the control or in the other sand variants (sand without the herbicide, and sand 14 months after herbicide application). Additionally, plants grown in all sand variants appeared light green, while those in the control were dark green (Figs. 3 and 4).

**Table 2:** The average specific growth rate (*R*) and the percentage inhibition of growth rate (*IR*) for the number of plants, number of fronds, and total frond area (cm^2^) (parameters shown in Fig. 5). The following were presented: the Swedish Standard (SIS) medium (control), as well as variants with water extracts of sand, sand mixed with soil, and soil alone. Each was tested both without and with herbicide. Measurements were taken at 1 day and 14 months after herbicide application. The tables display the arithmetic means with standard deviations (±). Abbreviations: + H—samples treated with herbicide; 1d—one day after application; 14 m—fourteen months after herbicide application. Asterisks (*) indicate statistically significant differences between the water extracts from tested herbicide-treated substrates and the control (Swedish Standard (SIS) medium). The Kruskal–Wallis ANOVA followed by a multiple comparison of mean ranks (Kruskal–Wallis test for multiple independent samples) was used; significance level *α* = 0.05. The results of this comprehensive statistical analysis are presented in Table S4—a supplementary Excel file

Parameter	Number of plants		Number of fronds		Total frond area (cm^2^)	
**Variants**	***R***	***IR ***(%)	***R***	***IR*** (%)	***R***	***IR*** (%)
SIS	0.255 ± 0.014	0.000	0.245 ± 0.015	0.000	0.260 ± 0.012	0.000
Sand	0.169 ± 0.018*	33.941 ± 6.891*	0.140 ± 0.011*	42.628 ± 4.587*	0.151 ± 0.013*	41.844 ± 5.023*
Sand + H 1d	0.172 ± 0.009*	32.749 ± 3.552*	0.140 ± 0.013*	42.589 ± 5.479*	0.115 ± 0.007*	55.669 ± 2.651*
Sand + H 14 m	0.164 ± 0.008*	35.602 ± 3.228*	0.138 ± 0.018*	43.508 ± 7.484*	0.153 ± 0.015*	41.017 ± 5.852*
Sand:soil	0.183 ± 0.017	28.526 ± 6.827	0.177 ± 0.020	27.543 ± 8.051	0.239 ± 0.027	8.245 ± 10.452
Sand:soil + H 1d	0.228 ± 0.012	10.750 ± 4.504	0.201 ± 0.012	17.946 ± 5.020	0.206 ± 0.014	20.627 ± 5.430
Sand:soil + H 14 m	0.185 ± 0.012	27.479 ± 4.562	0.180 ± 0.022	26.339 ± 9.130	0.202 ± 0.018	22.518 ± 6.785
Soil	0.220 ± 0.011	13.978 ± 4.384	0.198 ± 0.007	19.181 ± 3.052	0.254 ± 0.009	2.381 ± 3.462
Soil + H 1d	0.186 ± 0.024	27.113 ± 9.505	0.168 ± 0.013	31.259 ± 5.474	0.174 ± 0.021*	33.072 ± 7.978*
Soil + H 14 m	0.188 ± 0.022	26.213 ± 8.676	0.173 ± 0.011	29.179 ± 4.315	0.218 ± 0.017	16.166 ± 6.723

After 7 days of the experiment, only minor, statistically insignificant differences in the number of plants were observed between the different test variants. The fewest plants were found in the variant with sand 14 months after herbicide application, with a significantly lower specific growth rate of plant numbers compared to the sand:soil + H 1 d and soil-only variants. Slightly more plants were present in the sand-only variant, and the highest number was observed in the sand plus herbicide variant 1 day after herbicide application. All test variants had significantly fewer plants than the control (see Fig. 5, Table 2; Supplementary materials, Table S3).

In terms of frond area, there were no significant differences between the variants. The smallest frond area was recorded in the sand variant 1 day after herbicide addition, followed by sand alone, and then sand with glyphosate after 14 months. The frond area in all test variants was approximately half that of the control, and these differences were statistically significant. At the end of the study, all variants containing sand showed a statistically significant reduction in the growth rate of frond area compared to all variants with mixed sand and soil, soil alone, and soil with herbicide after 14 months (Fig. 5 and Table 2; Supplementary materials, Table S3). The number of fronds in all sand-based variants was similar (no significant differences) and about half the number found in the control. The differences between the control and the variants with and without herbicide were statistically significant. Additionally, the decline in the growth rate of frond number at the end of the experiment was significantly greater in all sand variants compared to the mixed sand and soil, soil alone, and soil with herbicide after 14 months (Fig. 5 and Table 2; Supplementary materials, Table S3).

The fresh weight of the plants was the lowest in the sand with glyphosate one day after application, amounting to approximately one-third of the control—this difference was statistically significant, also compared to sand:soil, soil alone, and soil + H 14 m variants. The next lowest was in the sand-only (no herbicide) treatment, with slightly less than half of the control’s fresh weight. The highest fresh weight was observed in the sand 14 months after herbicide treatment, exceeding half of the control. The dry weight of the plants was similar across all sand-based variants, remaining at the control level (no significant differences). The sand + H 1 d variant had the lowest water content percentage and the highest dry matter percentage, and this difference was statistically significant compared to the control (see Table 3; Supplementary materials, Table S4).

**Table 3:** *Lemna* test results: fresh weight (g), dry weight (g), water content (%), and dry matter content (%) of *L. minor* plants at the end of the research

Parameter	Fresh weigth (g)	Dry weight (g)	Water content (%)	Dry matter content (%)
**Variants**				
SIS	0.094 ± 0.024	0.002 ± 0.001	97.351 ± 0.319	2.649 ± 0.319
sand	0.043 ± 0.006	0.003 ± 0.001	93.825 ± 1.304*	6.175 ± 1.304*
sand + H 1d	0.027 ± 0.004*	0.002 ± 0.000	90.995 ± 1.804*	9.005 ± 1.084*
sand + H 14 m	0.052 ± 0.010	0.003 ± 0.001	95.066 ± 1.543	4.934 ± 1.543
sand:soil	0.066 ± 0.012	0.003 ± 0.001	95.493 ± 0.907	4.507 ± 0.907
sand:soil + H 1d	0.047 ± 0.010	0.003 ± 0.001	94.524 ± 2.272	5.476 ± 2.272
sand:soil + H 14 m	0.049 ± 0.008	0.002 ± 0.000	95.124 ± 1.173	4.876 ± 1.173
soil	0.093 ± 0.026	0.005 ± 0.002	94.142 ± 1.871	5.858 ± 1.871
soil + H 1d	0.037 ± 0.009*	0.003 ± 0.000	92.847 ± 1.122*	7.153 ± 1.122*
soil + H 14 m	0.090 ± 0.030	0.003 ± 0.000	95.982 ± 1.164	4.018 ± 1.164

*L. minor* plants in the control (Swedish Standard (SIS) medium) exhibited a typical appearance (Figs. 3 and 4) and showed a normal increase in the measured parameters (number and area of fronds, number of plants, fresh and dry weight, and root length). Among the reference variants without herbicide addition, the plants grew the least in the sand-only variant. In comparison to the control plants grown in the SIS medium, the increase in the parameters studied in *L. minor* plants grown in the sand extract without the herbicide was approximately 50% smaller (Fig. 5 and Tables 2 and 3; Supplementary materials, Tables S3–S4).

#### Microtox

**Fig. 6:**
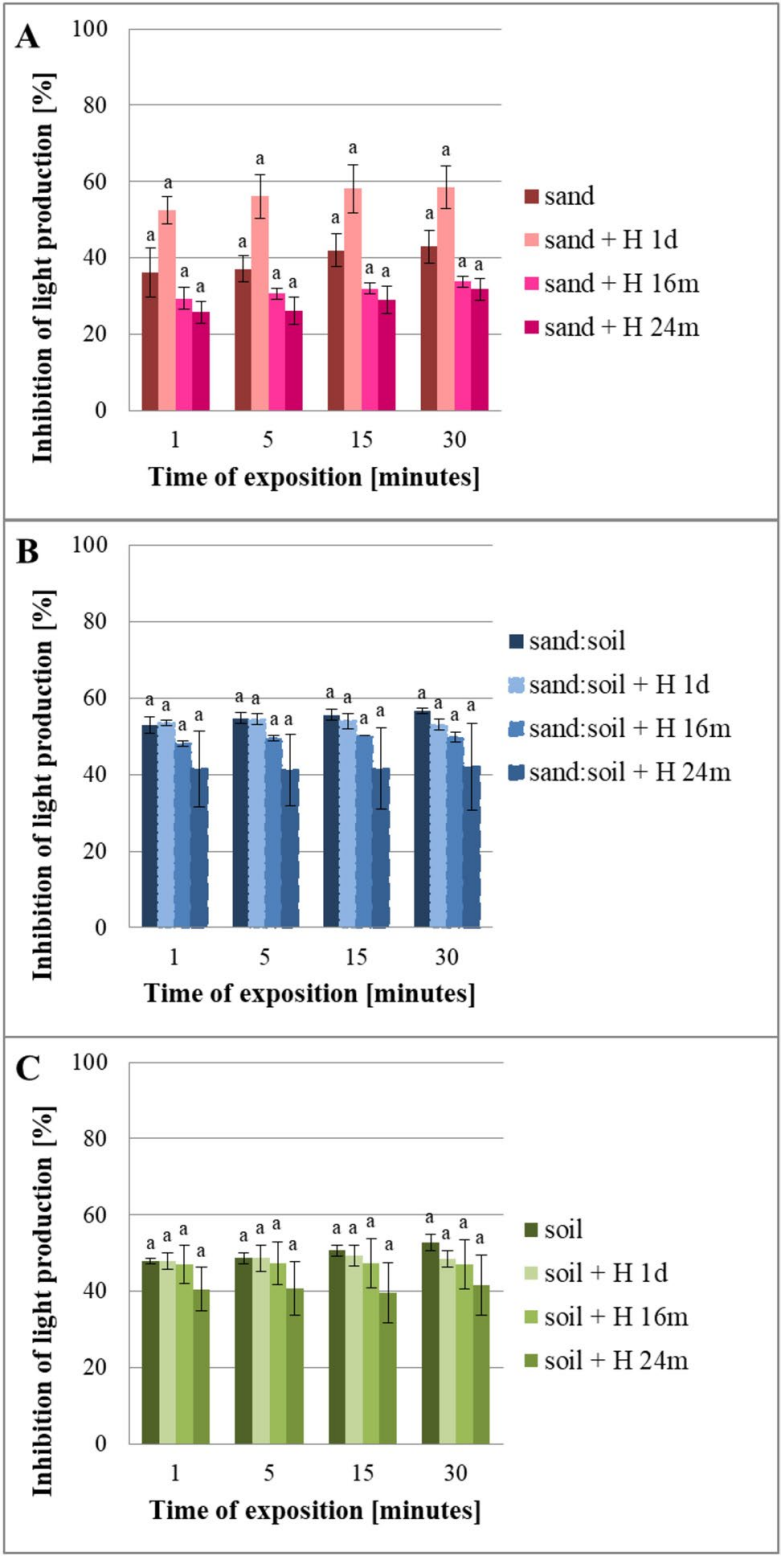
Inhibition of light production in *V. fischeri* bacteria after 1, 5, 15, and 30 min of exposure to extracts from the substrates (at 100% concentration): **A** sand, **B** sand with soil, and **C** soil. Each was tested both without and with herbicide, relative to the control (2% NaCl solution). Measurements were taken at 1 day, 16 months, and 24 months after herbicide application. Abbreviations: + H—samples with herbicide; 1d—one day; 16 m—sixteen months; 24 m—twenty-four months after herbicide application. The charts display arithmetic means with standard deviations. No statistically significant differences were observed (Kruskal–Wallis ANOVA test followed by a multiple comparison of mean ranks for all samples—Kruskal–Wallis test for multiple independent samples; significance level *α* = 0.05). In addition, the results of the statistical analysis (Kruskal–Wallis test for multiple independent samples) are presented in Table S5—a supplementary Excel file

Although the results of the Microtox biotest were not statistically significant due to an insufficient number of repetitions, some trends were observed, as shown below. The greatest inhibition of *V. fischeri* luminescence was seen in the sand variant with glyphosate after one day of herbicide addition—approximately 60%. The sand variant without glyphosate showed about 40% inhibition. Lower levels of bacterial luminescence inhibition were observed in the sand variants after longer periods since herbicide application (16 and 24 months), with inhibition levels around 25–30%. In all these variants, the inhibition of light production increased very slightly with the incubation time of the bacteria with the samples. The results for the sand variants were mostly similar to those obtained for sand mixed with soil and for the soil itself. However, the greatest inhibition of bacterial luminescence among all tested samples was observed in the sand sample one day after herbicide application (Fig. 6; Supplementary materials, Table S5). No statistically significant differences were found between the studied variants. In the Microtox test, the level of *V. fischeri* fluorescence in the control remained consistently at 100%.

#### Glyphosate and AMPA content

**Fig. 7:**
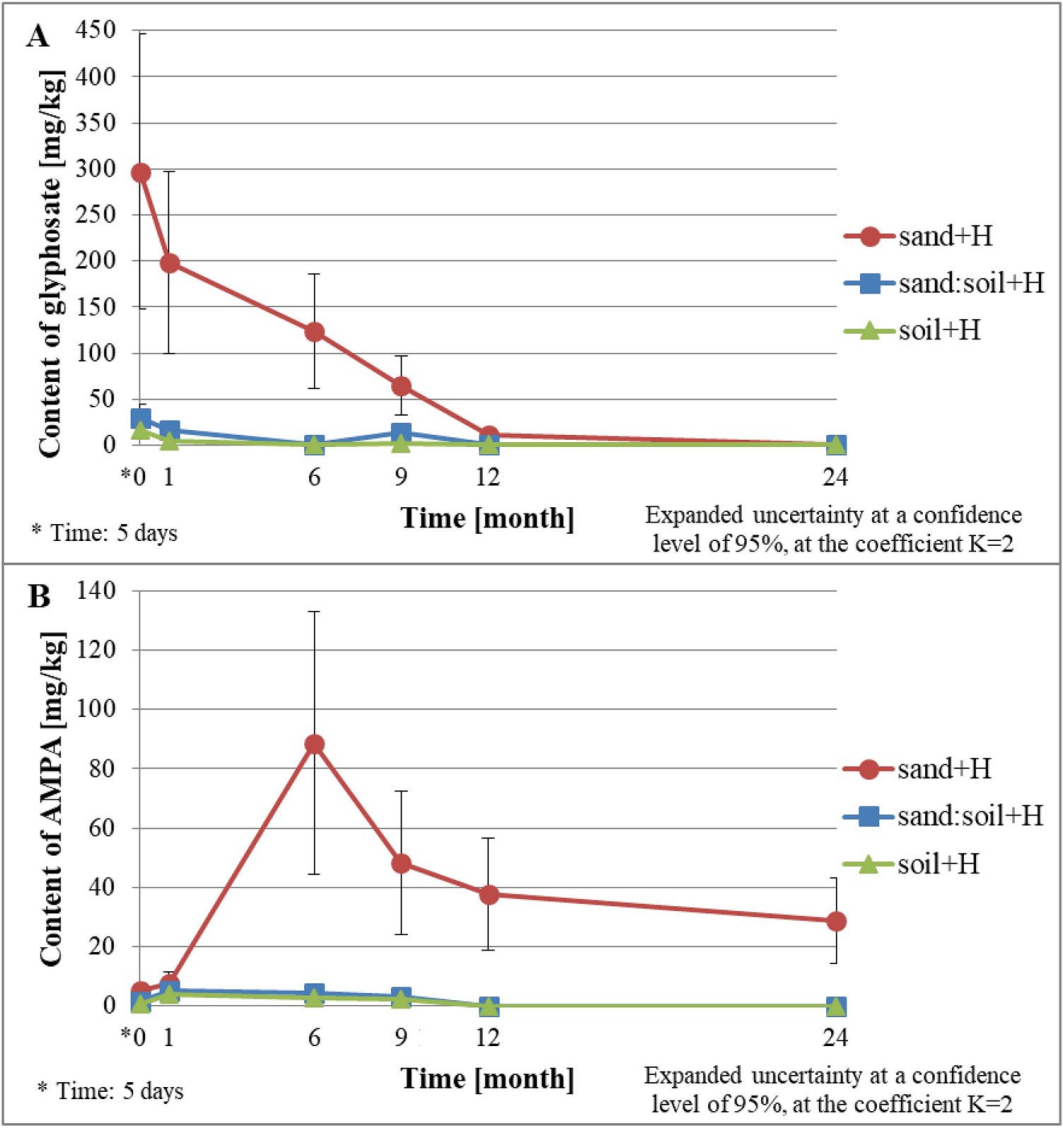
Content of glyphosate (**A**) and AMPA (**B**) in the tested substrates (sand, sand with soil, and soil) after 5 days (0 months), 1, 6, 9, 12, and 24 months following herbicide application (mg/kg). Abbreviations: + H—samples with herbicide. Since each variant was tested with individual mixed samples, no statistical analyses were performed. The graphs from the chemical analyses display results with expanded uncertainty at a 95% confidence level, using a coefficient *K* = 2

The highest glyphosate content was found in the sand both after 5 days and after 1, 6, 9, 12, and 24 months from the herbicide application to the substrates. For sand alone, a significant reduction in glyphosate levels was only observed after 12 months (Fig. 7A). Regarding AMPA, its highest concentration among all tested substrates was consistently found in the sand-only variant. During the first month, the AMPA content in the tested medium remained low. In the case of sand, a significant increase in AMPA levels was observed six months after the herbicide was applied. Even 24 months postapplication, the high AMPA concentration in sand was maintained (Fig. 7B).

#### Phosphorus and nitrogen content

**Fig. 8:**
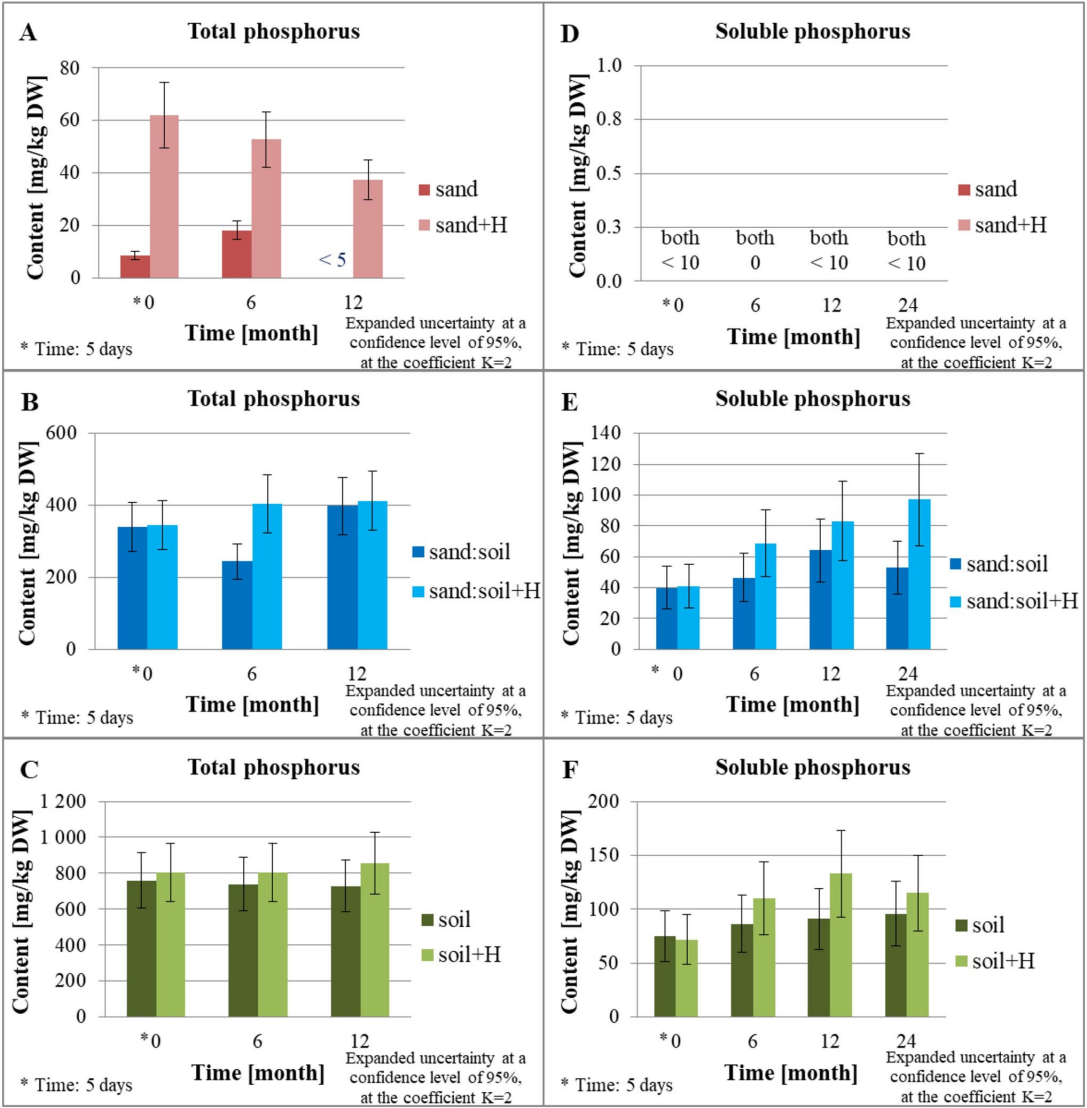
Content of total phosphorus (**A–C**) and soluble phosphorus (**D–F**) (mg/kg DW) in the tested and control substrates: sand (**A**, **D**), sand with soil (**B**, **E**), and soil (**C**, **F**). Measurements were taken at 5 days (0 months), 6 months, 12 months, and 24 months (the latter only for soluble phosphorus) after herbicide application. The same measurement times were used for control substrates without herbicide. Abbreviations: + H—samples with herbicide. Since individual mixed samples were tested for each variant, no statistical analyses were performed. The graphs from the chemical analyses display results with expanded uncertainty at a 95% confidence level, using a coefficient *K* = 2

The lowest total phosphorus content was observed in the sand variant. In the case of sand, the control had a lower total phosphorus content than the herbicide variant at all tested time points. Over time, the phosphorus content in the sand of the herbicide variant gradually decreased, but unlike the control, it was not completely depleted after 12 months (Fig. 8A). In the sand-only variants, no soluble phosphorus was detected at any of the tested time points (Fig. 8D).

**Fig. 9:**
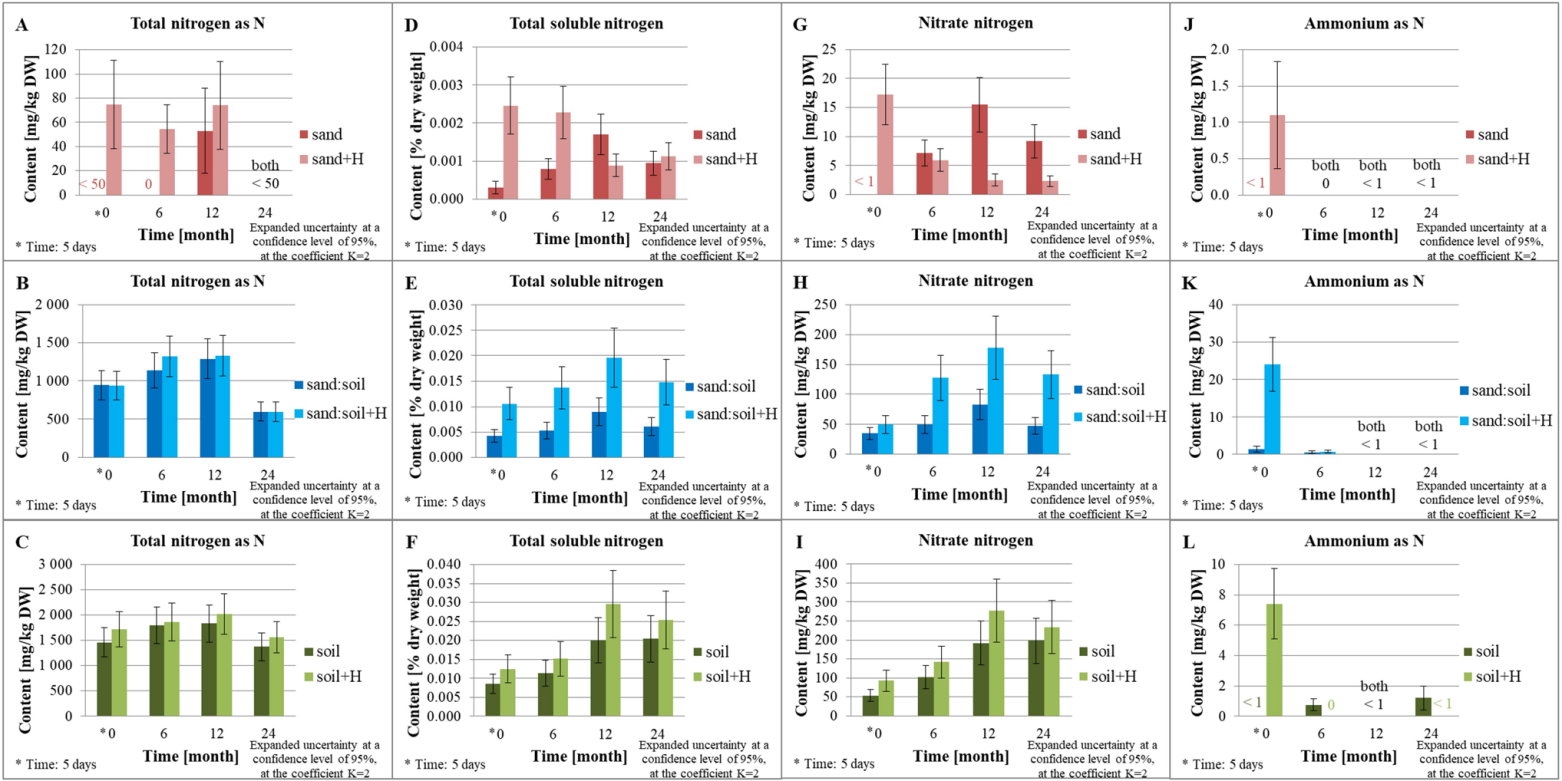
Content of total nitrogen as N (mg/kg) (**A–C**), total soluble nitrogen (% dry weight) (**D–F**), nitrate nitrogen (**G–I**), and ammonium as N (**J–L**) (mg/kg dry weight) in the tested and control substrates: sand (**A**, **D**, **G**, **J**), sand with soil (**B**, **E**, **H**, **K**), and soil (**C**, **F**, **I**, **L**). Measurements were taken at 5 days (0 months), 6 months, 12 months, and 24 months after herbicide application. The same measurement times were used for control substrates without herbicide. Abbreviations: + H—samples with herbicide. Since each variant was tested with individual mixed samples, no statistical analyses were performed. The graphs from the chemical analyses display results with expanded uncertainty at a 95% confidence level, using a coefficient *K* = 2

The lowest total nitrogen content was observed in the sand variants. In the case of sand, the total nitrogen content was lower in the control than in the herbicide-treated variant at the tested time points. The total nitrogen content in sand varied across different time points in the herbicide-treated variant. At 0, 6, and 24 months, no total nitrogen was detected in the control, and after 24 months, no total nitrogen was detected in the herbicide-treated variant (Fig. 9A).

In the case of total soluble nitrogen, the lowest levels were observed in the sand variants. Among the sand variants, the control showed a gradual increase in total soluble nitrogen content up to 12 months, followed by a decrease after 24 months. In the sand variant where the herbicide was applied, a gradual decrease in total soluble nitrogen was observed over time, with levels at 12 and 24 months being similar (Fig. 9D).

The lowest nitrate nitrogen content was observed in the sand variants. In this medium, the control showed a gradual increase in nitrate nitrogen levels over 12 months, followed by a decrease after 24 months. In the sand variant with the herbicide, a gradual decrease in nitrate nitrogen content was observed over time (Fig. 9G). In the sand variants, a small amount of ammoniacal nitrogen was detected in the herbicide-treated variant only 5 days after application. No ammoniacal nitrogen was detected in the control (Fig. 9J).

#### Sand—summary

The results obtained for the variants with sand alone (the substrate with the lowest organic matter content) indicate that:

- The toxic effects of undecomposed glyphosate and AMPA on organisms incubated in sand variants lasted up to 24 months.
- Glyphosate takes more than 12 months to break down in sand, and AMPA takes more than 24 months.
- Adding glyphosate to sand resulted in an increase in the amount of phosphorus and nitrogen compared to the control substrate without the herbicide, with this effect lasting up to 12–24 months.

### Substrate with medium organic matter content—sand mixed with soil

#### Phytotoxkit

In the herbicide-treated variant, where the plants grew on sand mixed with soil, the inhibition of *L. sativum* root growth during the first three months was similar to that observed in plants grown on sand with the herbicide (Fig. 1; Supplementary materials, Table S1). The toxicity index during this period was approximately 80% (Fig. 2; Supplementary materials, Table S2). From the sixth month onward, root length in the glyphosate-treated sand–soil variant was comparable to the control (Fig. 1; Supplementary materials, Table S1), and the toxicity index for this treatment ranged between 10 and 20% from 6 to 24 months (Fig. 2; Supplementary materials, Table S2). Statistical analysis showed significant differences in root length between the herbicide-treated variant (sand:soil + H) and the control (sand:soil) after day 1, as well as after 1, 3, 13, and 24 months—most notably between days 2 and 7 of the test (Figs. 1 and 2; Supplementary materials, Tables S1 and S2). For the herbicide-treated samples, significant differences in toxicity index between the sand:soil + H and sand + H variants appeared after 6 months, and for the soil + H variant after 13 months. These differences persisted up to 24 months in both cases (Fig. 2; Supplementary materials, Table S2).

In the Phytotoxkit test, the roots of the control *L. sativum* plants growing in a substrate with medium organic matter content—specifically, sand mixed with soil in a 1:1 ratio—were typically about 60–80 mm long at all tested time points. The differences in root length between plants grown in the sand–soil mixture and those in other control media were not statistically significant (Fig. 1; Supplementary materials, Table S1).

#### *Lemna* test

The *L. minor* plants grown in the sand–soil mixture—both in the absence of herbicide and in the variants with herbicide applied (1 day and 14 months after glyphosate addition)—exhibited smaller growth compared to the control, but larger than in the sand-only variants across all study endpoints (Figs. 3 and 5 and Tables 2 and 3; Supplementary materials, Table S3 and S4). One day after herbicide application in the sand–soil mixture, the plants showed significantly shortened roots (~ 1 cm long) and chlorosis, which were not observed in the control or in other sand–soil variants (without herbicide and with herbicide after 14 months). Nonetheless, the plants in these variants remained as dark green as those in the control (Figs. 3 and 4).

After 7 days of the test, the fewest plants were observed in the variants with sand and soil without herbicide, as well as in the glyphosate-treated sand and soil after 14 months. Slightly more plants were found in the variants with a mixture of sand and soil one day after herbicide application. The number of plants was lower than in the control (differences were not statistically significant) and slightly higher than in the sand-only variants. Notably, the sand:soil + H 1 d variant had a statistically higher specific growth rate in the number of plants compared to the sand-only variants (Fig. 5 and Table 2; Supplementary materials, Table S3). The smallest frond area was observed in the variant with sand and soil 14 months after the addition of glyphosate (statistically significant compared to the control and the sand:soil variant). The next smallest was in the mixture of sand and soil one day after herbicide application, followed by the medium organic matter content substrate without herbicide (not statistically significant compared to the control). The specific growth rate of frond area at the end of the study was higher in all sand:soil variants than in the plants grown in sand-only variants (statistically significant differences) and similar to those in the corresponding soil-only variants (Fig. 5 and Table 2; Supplementary materials, Table S3). All variants with sand:soil had significantly fewer fronds than the control (the lowest in the variants without herbicide and with glyphosate after 14 months; slightly higher in the 1-day herbicide addition variant). The inhibition of the growth rate of frond numbers was significantly lower in all sand:soil variants compared to all sand-only variants. Additionally, the inhibition of frond number growth in the sand:soil + H 1 d variant was significantly lower than in the soil + H 1 d variant (Fig. 5 and Table 2; Supplementary materials, Table S3).

Fresh plant weight was lowest in the variants with sand and soil both 1 day and 14 months after herbicide application (approximately half of the control). It was slightly higher in the variant with a mixture of sand and soil without herbicide. The dry weight of plants in all sand and soil variants was similar to that of the control. Although some differences were observed, they were not statistically significant for either fresh or dry matter, both among the tested variants and in comparison to the control. The same applies to the percentage of water and dry matter content (Table 3; Su﻿pplementary materials, Table S4).

Among the reference variants without herbicide addition, *L. minor* plants grown in sand mixed with soil showed an average increase in the measured parameters—though this increase was weaker than that of the control plants cultivated in Swedish Standard (SIS) medium by approximately (Figs. 3, 4, and 5 and Tables 2 and 3; Supplementary materials, Tables S3–S4).

#### Microtox

The inhibition of the *V. fischeri* bacteria’s glow intensity in the variants with sand and soil was quite similar and remained constant over time, comparable to the variants with only sand or only soil. For the variants with sand and soil—without herbicide, with herbicide after 1 day, and with herbicide after 16 months—the inhibition of light production was approximately 50%. In contrast, for the variant with sand and soil treated with glyphosate, the inhibition after 24 months was about 40%. Similar results were observed in comparable variants with soil alone (Fig. 6; Supplementary materials, Table S5). No statistically significant differences were found between the studied variants, likely due to the small number of replicates.

#### Glyphosate and AMPA content

Five days after adding the herbicide to the substrates, as well as after 1, 6, 9, 12, and 24 months, the average glyphosate content was observed in the sand mixed with soil, in comparison to other types of substrates. Nearly complete disappearance of glyphosate in the sand–soil mixture was observed six months after the herbicide was applied to the substrates (Fig. 7A). The average AMPA content was observed in the variant with sand mixed with soil, compared to other variants. In this variant, the AMPA levels remained low at all tested time points (Fig. 7B).

#### Phosphorus and nitrogen content

The average total phosphorus content was observed in the variant with sand mixed with soil (Fig. 8B). In the control variants with sand mixed with soil, the level of total phosphorus fluctuated over time, while in the glyphosate-treated variant, the phosphorus content slightly increased with time (Fig. 8B). The differences observed between the control and herbicide-treated variants with sand mixed with soil were less pronounced than those seen in the corresponding variants with sand alone (Fig. 8A, and B).

In the case of soluble phosphorus, its average content at specific time points was observed in sand mixed with soil. For this medium, five days after the herbicide application, the glyphosate-treated variants contained similar amounts of soluble phosphorus as the control groups. Over time, the soluble phosphorus content in the sand and soil mixtures, as well as in soil-only samples, gradually increased in both the control and herbicide-treated variants. However, a faster increase and higher levels of soluble phosphorus were observed in the herbicide-treated variants. This trend continued for up to 24 months (Fig. 8E).

The average total nitrogen content was observed in the variants with sand mixed into the soil. In the sand–soil variants, both in the control and herbicide-treated samples, the total nitrogen level increased over the first 12 months, then decreased by about half after 24 months. Faster growth and higher total nitrogen levels were detected in the glyphosate-treated medium (Fig. 9B).

In the variants with sand mixed with soil, the mean total soluble nitrogen was detected. In the sand-and-soil variants, the content of total soluble nitrogen gradually increased over time in both the control and the glyphosate-treated variants up to 12 months. However, a faster increase and higher levels of total soluble nitrogen were observed in the herbicide-treated variant. After 24 months, the total soluble nitrogen content slightly decreased in both the control and the herbicide-treated samples compared to the 12-month time point measurements (Fig. 9E).

In the case of sand mixed with soil, the average nitrate nitrogen content was measured. In this medium, nitrate nitrogen levels gradually increased in both the control and glyphosate-treated variants up to 12 months, then decreased after 24 months. Faster growth and higher nitrate nitrogen content were observed in the post-herbicide variant (Fig. 9H). Regarding ammonium nitrogen, several times higher levels were detected at time zero in the herbicide-treated variant—representing the highest ammoniacal nitrogen level among all tested—compared to the control. After 6 months, ammoniacal nitrogen levels in both variants were similarly low (Fig. 9K).

#### Sand mixed with soil—summary

The obtained results for the variants with sand mixed with soil (substrate with medium organic matter content) indicate that

- The toxic effects of undecomposed glyphosate and AMPA on organisms incubated in sand mixed with the soil treated with the herbicide last for approximately 3 months, based on biotest results.
- Rapid decomposition of glyphosate and AMPA occurs within a few days after herbicide application, but these substances are detectable for up to 9 months in sand mixed with the soil variants.
- Adding glyphosate to sand mixed with soil increased the levels of phosphorus and nitrogen compared to the control substrate without glyphosate, with these elevated levels persisting for 12–24 months. However, these differences were not as pronounced as in sand treated directly with the herbicide.

### Substrate with the highest content of organic matter— soil

#### Phytotoxkit

In the case of the soil-only herbicide-treated variant (soil + H), the inhibition of *L. sativum* root growth over a period of up to one month was similarly low to that observed in the other two substrate types—approximately 80% toxicity index (Figs. 1 and 2; Supplementary materials, Tables S1 and S2). After three months, the inhibition of root growth in the herbicide-treated soil had decreased by half, with a toxicity index of about 40%. Up to the third month, the differences in root length between the herbicide-treated variant and the control were statistically significant, typically between days 2 and 7 of the test (Figs. 1 and 2; Supplementary materials, Tables S1 and S2). From the 6th to the 13th month, the growth of *L. sativum* roots in soil treated with the herbicide was generally similar to the control. However, during the initial days, a slight stimulation of root growth—about 10–20%—was observed compared to the control, followed by a minor inhibition, with a toxicity index of approximately 20% (Figs. 1 and 2; Supplementary materials, Table S1 and S2). For the herbicide-treated variants, statistically significant differences in the toxicity index between soil (soil + H) and sand (sand + H) began to appear after 3 months, while in the sand with soil variant (sand:soil + H), these differences emerged after 13 months. In both cases, the differences persisted up to 24 months (Fig. 2; Supplementary materials, Table S2).

After 24 months, the growth of *L. sativum* roots in the soil + H variant was better than the control throughout all 7 days of the Phytotoxkit test. On the first and second days, root growth was significantly greater than in the control (60–180%). From the 3rd to the 7th day, root growth remained approximately 30–50% higher than in the control, with the difference gradually decreasing (Fig. 2; Supplementary materials, Table S2). The observed differences in root length between the herbicide variant and the control were statistically significant at the 24-month time point, from day 1 to day 7 of the test (Figs. 1 and 2; Supplementary materials, Tables S1 and S2).

In the Phytotoxkit test, the roots of the control plants growing in pure soil (substrate with the highest content of organic matter) reached lengths similar to those in the other control substrates—typically about 60–80 mm—at all tested time points. The differences in root length of *L. sativum* plants grown in soil compared with other control substrates were not statistically significant (Fig. 1; Supplementary materials, Table S1).

#### *Lemna* test

The *L. minor* plants in the herbicide-treated soil variants exhibited smaller growth compared to the control, while in the soil variant without herbicide, plant growth was similar to that of the control. Generally, plant growth in soil variants was larger than in sand and sand–soil variants (Figs. 3 and 5 and Tables 2 and 3; Supplementary materials, Tables S3–S4). In the soil variant with herbicide, after 1 day, strongly shortened roots (~ 1 cm long) and chlorosis were observed in the plants—effects not seen in the control or other soil variants (soil without herbicide and 14 months after glyphosate application). Plants in these variants remained as dark green as those in the control (Figs. 3 and 4).

After 7 days of the test, the fewest plants were observed in the variants with soil and herbicide (14 months and 1 day after glyphosate application), with slightly more plants in the soil without herbicide. The number of plants was lower than in the control (differences were not statistically significant) and comparable to the sand and soil variants (Fig. 5 and Table 2; Supplementary materials, Table S3). The smallest frond area was observed in the soil + H variant after 1 day (significantly smaller than both the control and the soil-only variant), followed by the soil + H variant after 14 months. The specific growth rate of frond area in all soil variants at the end of the experiment was higher than in all sand variants (most differences were statistically significant) and higher than in the sand–soil variants (mostly not significant) (Fig. 5 and Table 2; Supplementary materials, Table S3). The number of fronds in the herbicide-treated soil variants after 1 day and 14 months was about one-third of that in the control, while in the soil without herbicide, it was approximately one-fifth lower than in the control (statistically significant differences). The specific growth rate of the number of fronds at the end of the study was similar in the soil variants and those with sand and soil (no statistically significant differences), and it was higher than in all sand variants (the differences for soil and soil + H after 14 months were statistically significant) (Fig. 5 and Table 2; Supplementary materials, Table S3).

The fresh weight of the plant was lowest in soil treated with glyphosate after 1 day (about half of the control, with a statistically significant difference). In the variants without the herbicide and in soil with the herbicide after 14 months, it was at the control level (no significant differences). The percentage of water content in the soil + H 1 d variant was significantly different from the control. The dry weight of the plants was at the control level in all soil variants (no statistically significant differences). The dry matter content across soil variants and the control was not significantly different (Table 3; Supplementary materials, Table S4).

Among the reference variants without herbicide addition, *L. minor* plants in the soil-only variant showed the greatest increase in the measured parameters. Plant growth in this variant was either comparable to the control (Swedish Standard (SIS) medium) or slightly lower for some parameters. The only notable difference was in the number of fronds in the soil-only variant, which was significantly higher than in the control (Figs. 3, 4, and 5 and Tables 2 and 3; Supplementary materials, Tables S3–S4).

#### Microtox

The inhibition of fluorescence intensity in *V. fischeri* bacteria was quite similar and remained constant over time in the soil-only variants and was comparable between the sand-only and sand–soil variants. In the soil-only variants without herbicide, as well as those with herbicide after 1 day and after 16 months, light production was inhibited by approximately 50%. Conversely, in soil with herbicide after 24 months, inhibition was around 40%. Similar results were observed in the analogous sand and soil variants (Fig. 6; Supplementary materials, Table S5). No statistically significant differences were found between the studied variants, likely due to the limited number of repetitions.

#### Glyphosate and AMPA content

The lowest glyphosate content was found in the soil after 5 days, as well as at 1, 6, 9, 12, and 24 months following the herbicide application to the substrate (Fig. 7A). The almost complete disappearance of glyphosate in the soil was observed 6 months after application. The lowest AMPA content was consistently observed in the soil-only variant. The AMPA levels remained low in this variant across all tested time points (Fig. 7B).

#### Phosphorus and nitrogen content

The highest total phosphorus content was observed in the soil-only variants (Fig. 8C). The phosphorus levels remained consistent over time in these variants. Additionally, the soil treated with the herbicide showed higher phosphorus content compared to the control (Fig. 8C).

In the soil-only variants, the highest soluble phosphorus content was observed at specific time points. Five days after the herbicide application, the glyphosate-treated soil variants contained similar amounts of soluble phosphorus as the controls. Over time, the soluble phosphorus content in the soil-only variants gradually increased in both the control and herbicide-treated groups. However, the increase was faster and the levels higher in the herbicide variants. This trend continued for up to 12 months, but after 24 months, slightly lower levels of soluble phosphorus were observed in these variants compared to those at 12 months (Fig. 8F).

The highest total nitrogen content was observed in the soil-only variants. In both the control and herbicide-treated soil-only variants, the total nitrogen levels increased over time up to 12 months and then slightly decreased after 24 months. Faster growth and higher total nitrogen levels were detected in the herbicide-treated substrate (Fig. 9C).

In the case of total soluble nitrogen, the highest levels were observed in the soil-only variants. Over time, the total soluble nitrogen content gradually increased in both the control and herbicide-treated variants up to 12 months. However, a significantly faster increase and higher levels were observed in the herbicide-treated variant. After 24 months, in the sand mixed with soil—both in the control and herbicide-treated samples—the total soluble nitrogen content slightly decreased from the levels at 12 months (Fig. 9F).

The highest nitrate nitrogen content was observed in the soil-only variants. In the soil, nitrate nitrogen levels gradually increased in the control and herbicide variants up to 12 months, then decreased after 24 months. Faster growth and higher nitrate nitrogen levels were detected in the herbicide variants (Fig. 9I). In the pure soil variants, at time zero, ammonium nitrogen was detected only in the herbicide-treated variants, while after 6 and 24 months, it was present only in the control, but at much lower levels (Fig. 9L).

### Soil—summary

The results obtained for the soil-only variants (substrate with the highest organic matter content) indicate that

- The toxic effects of undecomposed glyphosate and AMPA on organisms incubated in soil treated with the herbicide last for approximately three months, based on biotest results.
- Rapid decomposition of glyphosate and AMPA occurs within a few days after herbicide application; however, these substances remain detectable for up to nine months.
- Adding the herbicide to the soil increased the levels of phosphorus and nitrogen compared to the control substrate without the herbicide, with this effect lasting up to 12–24 months. Nonetheless, these differences were less pronounced than in the herbicide-treated sand and were also slightly smaller compared to the sand–soil variant.

## Discussion

Glyphosate, a widely used herbicide, and its primary degradation product, aminomethylphosphonic acid (AMPA), are well-studied substances that can have significant environmental and ecological impacts (e.g., Duke 2020; Padilla and Selim 2020; Singh et al. 2020a, b). Although there are studies suggesting that the degradation of glyphosate and AMPA in soil may affect organisms living in the soil environment, particularly microorganisms (e.g., Gomes et al. 2014; Mérey et al. 2016; Klátyik et al. 2023; Li et al. 2025), a direct link between the concentrations of these compounds and toxicity to a wide range of organisms requires further, detailed research. It should also be noted that the results of such studies often depend on the context of environmental conditions and the research methods used.

In this study, we examined how long glyphosate and AMPA decompose in substrates with varying organic matter content and when these substances cease to be toxic to organisms. To provide a comprehensive assessment, we employed a combination of ecotoxicological testing using biotests and chemical analyses of the substrates. This multifaceted approach to evaluating substrate toxicity—using a battery of biotests alongside chemical analyses—has proven useful, as demonstrated in the work of Wierzbicka et al. (2015). We also investigated whether glyphosate spraying could increase nitrogen and phosphorus levels in substrates with different organic matter contents, as a result of the decomposition process. Of particular interest are substrates with low organic matter content, such as railway areas, where high doses of glyphosate are often applied (Bemowska-Kałabun et al. 2021). Although combining ecotoxicological studies with chemical analyses under controlled laboratory conditions may not reveal all environmental implications, this approach provides a valuable basis for discussing the potential environmental effects of high glyphosate doses.

### The degradation of glyphosate and AMPA— an overview

The degradation time of glyphosate varies widely and depends on factors such as soil type, temperature, humidity, pH, organic matter content, clay content, exchangeable ions, and the presence of microorganisms (Schuette 1998; Safety Data Sheet Roundup 360 SL 2010; Mérey et al. 2016; Duke 2020; Padilla and Selim 2020; Singh et al. 2020a, b). Glyphosate is relatively immobile in the soil, primarily due to its strong adsorption to soil particles. One of the key factors influencing the amount of glyphosate adsorbed is the presence of phosphate in the soil. Because of the phosphonic acid residues in its structure, glyphosate readily binds to clay minerals and hydrated oxides in soil. It competes with inorganic phosphorus for binding sites. As the literature indicates, phosphate-induced effects on glyphosate mobility appear to be dependent on factors such as soil type (Schuette 1998; Haney et al. 2000; Safety data sheet Roundup 360 SL 2010; Duke 2020; Padilla and Selim 2020; Singh et al. 2020a, b).

Glyphosate primarily degrades through microbial activity, which is the main pathway for its breakdown in the soil. Its dissipation occurs rapidly at first. The rate of glyphosate degradation depends on soil conditions, including soil type and the presence of microorganisms. Microbial processes dominate its breakdown, with hydrolysis and oxidation serving as key pathways. This biological degradation occurs under both aerobic and anaerobic conditions, carried out by various soil bacteria (Cerdeira and Duke 2006; Dick et al. 2010; Lane 2011; Lane et al. 2012a, b; Mérey et al. 2016; Wimmer et al. 2023; Duke 2020). Microorganisms capable of degrading glyphosate include, for example, *Achromobacter* sp., *Comamonas odontotermitis*, *Ochrobactrum intermedium* and, *Pseudomonas* sp. (Kishore and Jacob 1987; Gimsing et al. 2004; Singh et al. 2020b). Glyphosate can also degrade abiotically via interactions with soil minerals like manganese oxides, which cleave the C-P bond, releasing orthophosphate. This process is slower than microbial degradation. Glyphosate strongly adsorbs to soil particles, limiting its bioavailability. Only 0.02–0.13% of glyphosate in soil is bioavailable, which reduces its leaching risk. Adsorption is influenced by soil pH, organic matter, and clay content (Barrett, McBride 2005; Nguyen et al. 2018; Wimmer et al. 2023).

The main breakdown product of glyphosate in plants, soil, and water is AMPA (aminomethylphosphonic acid), which results from the hydrolysis of the herbicide and is a product of microbial degradation of glyphosate. The pathway leading to AMPA involves C-N cleavage by a flavoprotein oxidoreductase. Simultaneously, glyoxylic acid is formed. Another pathway, involving the cleavage of the C-P bond with inorganic phosphate and sarcosine, has also been observed in isolated soil bacteria in the absence of phosphate. In this pathway, the degradation product is glycine. The degradation product here is glycine (Schuette 1998; Giesy et al. 2000; Gimsing et al. 2004; Peruzzo et al. 2008; Rampazzo 2009). AMPA is more persistent than glyphosate, with half-lives ranging from about two weeks to even 250 days or more, depending on soil conditions. Its slower degradation is attributed to kinetic sorption, which limits its bioavailability. AMPA is biologically degraded in soils, but at a slower rate compared to glyphosate. Its dissipation is enhanced by microbial activity, particularly in soils with higher organic carbon availability. Similar to glyphosate, AMPA can undergo abiotic degradation via interactions with soil minerals, though this process is less efficient (Giesy et al. 2000; Barrett, McBride 2005; Mérey et al. 2016; Duke 2020; Singh et al. 2020a; Wimmer et al. 2023).

### Decomposition of herbicide in substrates with varying organic matter content

In the substrates tested in this study, the slowest decline in the levels of glyphosate and AMPA (the main breakdown product of glyphosate) was observed in sand, which has a low organic matter content. AMPA was still detectable even 24 months after the application of herbicide. Conversely, the fastest reduction in glyphosate and AMPA levels occurred in agricultural soil—a substrate rich in organic matter—with nearly complete decomposition of these compounds observed within 6 to 9 months after herbicide application. These findings are consistent with those reported by other researchers, as shown below. It is worth noting that the results in this study were comparable to many field studies despite the experiment being conducted under controlled laboratory conditions, which eliminated many variables typically present in the environment (e.g., changes in temperature, light intensity, varying precipitation, or the presence of diverse soil organisms). This fact further supports the view that conducting screening experiments in laboratory conditions before launching large-scale field studies can help predict what might happen in environmental settings and facilitate the interpretation of the results obtained. Of course, it is important to keep in mind the limitations of this type of research.

For example, the studies by Börjesson and Torstensson on railway substrates—with a low organic matter content—show that glyphosate is strongly adsorbed in railway embankments. After the application of 3 L/ha of Roundup Bio on the railway embankment, the half-life (DT_50_) of glyphosate and AMPA typically ranged between 2 and 5 months post-application; in some cases, these substances persisted even longer. The time required for 90% dissipation of the applied herbicide (DT_90_) was most often between 1 and 2 years. Both glyphosate and AMPA generally penetrate no deeper than 30 cm into the railway embankment, although they can sometimes reach depths of up to 60 cm (Bӧrjesson and Torstensson 2000; Torstensson 2001; Torstensson et al. 2005). For comparison, the work of Al-Rajab and Schiavon (2010) shows the distribution of glyphosate in three types of agricultural soils: silt clay loam, clay loam, and sandy loam. Differences were observed both in the kinetics of glyphosate degradation over time within the same soil type and between different soil types. The studies indicated that the rate of glyphosate mineralization was highest shortly after application and decreased over time. Glyphosate mineralization occurred most rapidly in clay soil and most slowly in sandy loam soil (Al-Rajab and Schiavon 2010).

Research by Börjesson, Torstensson, and Al-Rajab, along with Schiavon (Bӧrjesson and Torstensson 2000; Torstensson 2001; Torstensson et al. 2005; Al-Rajab and Schiavon 2010), aligns with the results of our experiment – specifically, that in substrates with low organic matter content (such as sand), glyphosate and AMPA decompose more slowly. In our study, we conducted a simplified experiment, assuming that the entire dose of glyphosate enters the soil. Additionally, environmental conditions in railway areas are much more extreme—such as higher temperatures and light intensity—than those in the laboratory. Despite these differences, the observed trends in the degradation of glyphosate and AMPA were consistent with existing literature data for areas with low organic matter content.

It is also worth noting that the presence of undegraded glyphosate in railway areas may promote the emergence of plants tolerant to this herbicide, as demonstrated by Bemowska-Kałabun et al. (2021) in *Geranium robertianum* L., which occurs in railway areas in northern Poland. This is especially true when, due to imperfect spraying, the full dose of herbicide does not reach the vegetation. Studies by Andersson et al. (2024) support this, indicating that the impact of glyphosate on vegetation diminishes significantly with increasing distance from the sprayed area. At less than 1 m, the effect on nontarget plants is likely, but beyond 1.5 m, it becomes unlikely (Andersson et al. 2024).

In turn, Giesy et al. (2000) conducted field studies on agricultural and forest soils (i.e., with high organic matter content). They found that the mean half-life of glyphosate in the tested media was 32 days. In forest soils, the DT_50_ ranged from 1.4 to 60 days after glyphosate application. In arable soils, the half-life varied from 1.7 to 197.3 days, but was typically less than 60 days (Giesy et al. 2000). In contrast, in loess soil in Argentina, the glyphosate DT_50_ was 6 days (Bento et al. 2019). These studies are consistent with our results, which showed the fastest glyphosate degradation occurring in soil with higher organic matter content.

Different studies indicate that AMPA has a much longer breakdown time than glyphosate. The half-life of AMPA ranges from 76 to 240 days (Giesy et al. 2000), and some studies report even up to 514.9 days (Singh et al. 2020a), with an average degradation time of approximately 145 days (Giesy et al. 2000). The appearance of AMPA in the soil depends on the rate of glyphosate mineralization and varies with the type of substrate. For example, in arable soils in Argentina, glyphosate concentrations ranged from 35 to 1502 μg/kg, while AMPA concentrations ranged from 299 to 2256 μg/kg (Aparicio et al. 2013). The studies by Al-Rajab and Schiavon (2010) demonstrated that in sandy loam soil and silt clay loam, the decomposition of glyphosate to AMPA was slower—both compounds were still detectable 80 days after application—compared to clay loam soil, where nearly 100% of glyphosate had broken down into AMPA within the same period. These findings are consistent with the results obtained in the present study. However, it is also important to remember that laboratory conditions do not fully replicate environmental conditions found in agricultural or forested areas. The results obtained primarily indicate a trend that emerges in substrates with high organic matter content. As mentioned earlier, the rate of glyphosate and AMPA degradation is influenced not only by the organic matter content but also by factors such as pH, moisture, and ion concentrations, which are highly diverse in more fertile environments. Additionally, a significant difference between field and laboratory conditions lies in the composition of soil microorganisms. Undoubtedly, in this type of laboratory research, it would also be valuable to identify which microorganisms are present in the tested substrates.

### The effects of glyphosate and AMPA on plants and microorganisms

Glyphosate inhibits 5-enolpyruvate-shikim-3-phosphate (EPSP), a key enzyme in the shikimate pathway found in plants, fungi, and microorganisms. Its inhibition results in the disruption of essential aromatic amino acid synthesis (tryptophan, phenylalanine, and tyrosine), leading to impaired protein production and cell death. Disruption of the shikimate pathway leads to oxidative stress and affects photosynthesis and other physiological processes. Glyphosate can decrease chlorophyll content in plants. This is due to the degradation or inhibition of chlorophyll biosynthesis. Glyphosate may indirectly prevent chlorophyll synthesis by decreasing the Mg content in leaves, which leads to reduced chlorophyll levels and a lower photosynthetic rate. Moreover, by inducing Fe deficiency, glyphosate may also inhibit the biosynthesis of δ-aminolevulinic acid (ALA), a key component of the chlorophyll biosynthetic pathway. Glyphosate can inhibit PSII activity, electron transport rate, and non-photochemical energy dissipation processes. It can also alter the activity of PSI and reduce NADH and NADPH levels (Kishore et al. 1992; Schuette 1998; Gomes et al. 2014; Duke 2020; Singh et al. 2020a). Another mechanism of glyphosate action is desiccation, i.e., dehydration of plant tissues. Closure of the stomata reduces respiration and causes disturbances in photosynthesis. Glyphosate predominantly moves through the phloem and, to a lesser extent, the xylem. Its effects include inhibited plant growth, chlorosis, necrosis, yellowing of leaves and shoots, and eventually the death of aboveground parts and roots (Adamczewski 2014; Rao 2014; Singh et al. 2020a; Bemowska-Kałabun et al. 2021).

AMPA, while less phytotoxic than glyphosate, still causes significant physiological stress, including reduced chlorophyll content and photosynthesis rates in plants, as well as oxidative stress. Like glyphosate, AMPA disrupts photosynthesis by decreasing chlorophyll levels and interfering with the shikimate pathway, which is essential for the production of aromatic amino acids. Specifically, AMPA inhibits chlorophyll biosynthesis by reducing glycine decarboxylase activity, leading to the accumulation of glycine and a deprivation of glutamate. Exposure to glyphosate and AMPA induces oxidative stress in plants, as evidenced by increased hydrogen peroxide levels and the activation of antioxidant defence systems. However, at high concentrations, these defences can be overwhelmed, resulting in cellular damage. It is also worth noting that AMPA is much more mobile than glyphosate salts and degrades more slowly in the soil (Amrhein et al. 1980; Kjær et al. 2005; Gomes et al. 2014; Duke 2020; Singh et al. 2020a; Bemowska-Kałabun et al. 2021). For aquatic plants, the effect of glyphosate and AMPA is similar to that observed in terrestrial plants—primarily because both share similar pathways of aromatic amino acid synthesis, which are inhibited by glyphosate (Villamar-Ayala et al. 2019; Singh et al. 2020b).

It is also worth remembering that, as confirmed by the studies of other researchers mentioned earlier and by the research presented in this manuscript, AMPA poses an additional threat to organisms due to its long persistence in the soil. The persistent presence of AMPA in the environment raises concerns about its long-term effects on plant biodiversity. Moreover, studies by Singkaew and Chaikaew (2026) indicate that AMPA is not only subject to long-term decomposition but also can accumulate in soils; subsequent applications of the herbicide strongly increase its content in the substrate. This potentially leads to reduced plant diversity and altered ecosystem services over time. That is why it is so important to also test high doses of glyphosate and AMPA in different substrates, as in our studies.

When it comes to microorganisms, the issue of glyphosate and AMPA toxicity is somewhat more complex. On the one hand, some microorganisms can utilize glyphosate as a phosphorus source (e.g., *Arthrobacter* sp. GLP-1, *Alcaligenes* sp. GL, *Pseudomonas pseudomallei* 22, and *Flavobacterium* sp. GD1), as well as a nitrogen or carbon source (e.g., *Ochrobactrum intermedium strain* Sq20, *Agrobacterium radiobacter* strain SW9, and *Achromobacter* sp. strain LW9). However, some microorganisms possess glyphosate-sensitive EPSPS enzymes, making them susceptible to glyphosate’s effects. Glyphosate may disrupt soil microflora—benefiting certain microorganisms while being toxic to others. Nonetheless, the bioavailability of glyphosate to specific soil microorganisms remains unknown (Duke 2020; Singh et al. 2020b). Glyphosate can reduce bacterial diversity but increase microbial activity in soils, particularly in areas with lower prior exposure to the herbicide. It exerts selective pressure on microbial communities, leading to species replacement and changes in community structure. AMPA, while more persistent in soil, is less bioavailable due to strong sorption, which limits its degradation and increases ecological risks. Glyphosate and AMPA can alter nitrogen cycling processes, affecting soil enzyme activity and microbial functionality (Andrighetti et al. 2014; Wimmer et al. 2023; Liu et al. 2025). As you can see, assessing glyphosate’s impact on soil microflora is highly complex.

Finally, it is also worth mentioning that the disruption of plant growth and soil microbial communities can have cascading effects on ecosystem services, such as nutrient cycling and habitat provision. In our view, the complexity of the observed phenomena related to the degradation of glyphosate and AMPA, as well as their impact on organisms, requires the simultaneous conduct of ecotoxicological studies and chemical analyses. We have demonstrated this approach in our research.

### Herbicide toxicity in substrates with varying organic matter content—ecotoxicological assessment

In this study, the rate of glyphosate and AMPA decomposition in substrates with varying organic matter content was correlated with the toxicity of these substances to test organisms (*L. sativum*, *L. minor*, and *V. fischeri*). The biotests and organisms used in this work have also been successfully employed by other researchers to assess the toxicity of glyphosate, including in environmental samples (e.g., Bonnet et al. 2007; Kumar and Han 2010; Piotrowicz-Cieślak et al. 2010; Sihtmäe et al. 2013; Sikorski et al. 2019; Tzvetkova et al. 2019).

It was shown that in sand (a substrate with low organic matter content), glyphosate and/or AMPA were the most toxic to the tested organisms and remained toxic for the longest duration, with the slowest decomposition of glyphosate and AMPA. Even after glyphosate decomposition, the substances in sand remained toxic to *L. sativum* plants. In substrates with low organic matter content, long-term toxicity is likely caused more by un-decomposed AMPA than by glyphosate itself. However, this possibility warrants further investigation—such as analyzing substrates with varying organic matter content after the addition of AMPA alone and compared with glyphosate toxicity. In contrast, the herbicide in the agricultural soil alone was toxic to the tested organisms only for a short period after the herbicide application, consistent with the rapid degradation of glyphosate and AMPA observed in substrates with higher organic matter. The substrate with medium content of organic matter—a 1:1 mixture of sand and soil—exhibited a slightly longer duration of glyphosate/AMPA toxicity to organisms after herbicide application compared to soil alone.

The most sensitive biotest used to assess the toxicity of the tested media was the Phytotoxkit test with *L. sativum* plants. In the studies by Piotrowicz-Cieślak et al. (2010) and Tzvetkova et al. (2019), *L. sativum* was also successfully used to investigate glyphosate toxicity. In the work of Piotrowicz-Cieślak et al. (2010), the Phytotoxkit biotest was employed, with the endpoint being root length inhibition, similar to our research. Piotrowicz-Cieślak et al. (2010) used the following glyphosate doses: 0, 1, 3, 7, 10, 40, 80, 120, 180, 240, 400, 750, 1000, 1500, 1700, and 2000 µM. In our study, we used 180,000 µg of glyphosate, which corresponds to 1064.65 µM. In the study by Piotrowicz-Cieślak et al. (2010), glyphosate was added to a reference soil composed of sand, vermiculite, and peat in a 1:0.3:1 volume ratio, which is a standard mixture provided by the Phytotoxkit manufacturer. Since the reference substrate used in the Phytotoxkit test contained both sand and peat (Piotrowicz-Cieślak et al. 2010), it can be compared to the substrate with a medium organic matter content used in our studies—namely, a 1:1 mixture of sand and soil. In the study by Piotrowicz-Cieślak et al. (2010), the glyphosate concentration that effectively inhibited root growth in 50% of the plants (EC_50_) for *L. sativum* was 30 µM. In our study, we observed a similar inhibition of root growth in *L. sativum* on substrates with medium organic matter content across different time points—1 day, 1 month, and 3 months after glyphosate application. Comparable results were seen in agricultural soil (which has the highest organic matter content), although three months after herbicide application, the substrate’s toxicity to plants was lower than in the analogous substrate with medium organic content. In the case of sand (which has the lowest organic matter content), the inhibition of root growth was much more pronounced, reaching nearly 100% in all test variants—1 day, 1 month, and 3, 6, 9, 13, and 24 months after glyphosate application. This could be due to the predominance of undegraded AMPA in the sand. The obtained results clearly show how important it is to assess toxicity for substrates of different organic matter content because substances such as glyphosate and AMPA will decompose at different rates in substrates of different organic matter content. It is also worth noting that in studies of this type, it would be best to independently examine the effects of AMPA itself and to use different doses of glyphosate and AMPA. In our studies, we focused solely on high doses of the herbicide, which are unlikely to occur under natural conditions. Although, as studies Singkaew and Chaikaew (2026) show, there may be situations where AMPA accumulates in the soil with successive herbicide applications. Nevertheless, studies using bioassays represent only a simplified model of what we might encounter in the environment.

Another interesting aspect of Piotrowicz-Cieślak et al. (2010) was the myo-inositol content in roots, used as an endpoint. Myo-inositol is a stress metabolite in plants, stimulated by drought and salinity (Piotrowicz-Cieślak et al. 2010; Sikorski et al. 2015). Herbicides like glyphosate have a desiccative effect on plants (Adamczewski 2014; Rao 2014; Piotrowicz-Cieślak et al. 2010; Singh et al. 2020a; Bemowska-Kałabun et al. 2021). In their research, Piotrowicz-Cieślak et al. (2010) suggested that plant responses to herbicides might involve mechanisms similar to those active under drought stress—that is, the level of myo-inositol in plants treated with glyphosate increased, but only when the herbicide was applied at very high concentrations (above 10–40 µM). Under stress conditions (e.g., changes in humidity and temperature), plants accumulate various compounds, including carbohydrates, and modify the content of soluble carbohydrates such as myo-inositol. These carbohydrates protect cellular structures and proteins and also play a role in signal transduction within plants (Adomas and Piotrowicz-Cieślak 2004; Sikorski et al. 2015). Piotrowicz-Cieślak et al. (2010) demonstrated that this mechanism is also involved in the plant stress response to glyphosate, which is an interesting finding. Similarly, Sikorski et al. (2015), who investigated the phytotoxicity of glyphosate in *Lupinus luteus* L. seedlings, reached comparable conclusions. It would be valuable in future studies to include myo-inositol levels as one of the endpoints in glyphosate ecotoxicological assessments, alongside other stress-related markers such as proline content, lipid peroxidation, or chlorophyll content.

A review paper by Duke (2020) reports that glyphosate is tightly bound primarily by nonorganic soil components. Glyphosate competes with phosphate for adsorption sites in the soil. Consequently, the presence of phosphate may lead to the remobilization of glyphosate and an increase in the toxicity of unbound glyphosate (Duke 2020). In our study, we did not observe such a phenomenon in test organisms incubated in agricultural soil. It is possible that the substrate was not sufficiently rich in phosphate to trigger this effect. Duke (2020) also notes that in very sandy soils with limited binding sites, glyphosate residues can cause toxicity to organisms—which aligns with the results of the biotests in this study, conducted in sand, i.e., a substrate with low organic matter content. On the other hand, Kostina et al. (2023) demonstrated that adding glyphosate to arable soil with low organic content and low biological activity resulted in a significant decline in soil biological activity. It is important to note that deterioration of soil quality can also impact the vegetation in areas where glyphosate is applied. Given the variety of factors influencing glyphosate toxicity in the substrate, it seems reasonable to employ both standardized biotests and models that simulate substrates with different levels of organic matter content—such as in the present study.

As reported by, for example, Duke (2020), one of the main decomposition products of glyphosate—AMPA—is slightly more mobile than glyphosate in soil, which contributes to its likelihood of being found in groundwater and surface water. Additionally, Bai and Ogbourne (2016) mention that the detection of glyphosate and AMPA in water is becoming increasingly common. According to Villamar-Ayala et al. (2019), the mobility and consequently the concentration of glyphosate and AMPA in water are primarily influenced by runoff and leaching into groundwater or surface water (depending on factors such as soil saturation, proximity to water bodies, and weather or climate conditions), as well as by bio- and photodegradation (which depend on the physicochemical properties of the water). In aquatic environments, glyphosate and AMPA can either be deposited in sediments or undergo degradation (Villamar-Ayala et al. 2019). In the work of Cederlund (2022), the author points out that when glyphosate is applied at a rate of 1800 g/ha to railways, both glyphosate and AMPA can reach the groundwater directly beneath the track. Cederlund (2022) also showed that concentrations of glyphosate occasionally exceed the EU groundwater quality standards. Therefore, we not only considered terrestrial plants when selecting bioassays for the study but also examined the toxicity of glyphosate in soil extracts to the aquatic plant *Lemna minor* and marine bacteria *Vibrio fischeri*. Both of these species have already been used in ecotoxicological assessments of preparations containing glyphosate. Thus, for example, a review paper by Villamar-Ayala et al. (2019) reports that glyphosate causes acute toxicity (mortality) in the macrophyte *L. minor* when applied at doses of 0.8 kg a.i./ha (according to Lockhart et al. 1989). The LC_50_ for glyphosate salt ranges from 16 to 46.9 mg a.i./L, while the LC_50_ for Roundup is 11.2 mg a.i./L (based on Cedergreen and Streibig 2005; Kumar and Han 2010; Vera et al. 2010). Acute toxicity (7-day EC_50_) occurs at glyphosate concentrations higher than 16 mg a.i./L (as reported by Cedergreen and Streibig 2005; Kumar and Han 2010). Additionally, Villamar-Ayala et al. (2019) provide information that for the bacterium *V. fischeri*, the LC_50_ for Roundup is 24.9 mg a.i./L, and for AMPA, the LC_50_ ranges from 50.5 to 166.5 mg a.i./L (after Tsui and Chu 2003; Bonnet et al. 2007).

In our study, the biotest using the water plant *L. minor* with soil extracts proved to be less sensitive than the Phytotoxkit. In this bioassay, the most reliable indicators of substrate toxicity shortly after herbicide application were observations of root length, chlorosis on the plants, and measurements of the plants’ fresh weight. The other parameters tested were less sensitive. Although *L. minor* was not the most sensitive bioindicator in our study, it has been effectively used in other research, such as in Kumar and Han (2010) and Sikorski et al. (2019), to assess glyphosate toxicity. The responses of *L. minor* plants to the herbicide observed in these studies (Kumar and Han 2010; Sikorski et al. 2019) were similar to those in the present work—glyphosate caused a reduction in plant growth rate. The aforementioned studies employed additional endpoints (e.g., chlorophyll *a* fluorescence and the content of chlorophyll *a*, *b*, and carotenoids) and demonstrated, among other things, that glyphosate impairs the synthesis of chlorophyll *a* and *b* in *L. minor* plants (Kumar and Han 2010; Sikorski et al. 2019). This aligns with our macroscopic observations—the appearance of chlorosis on *L. minor* fronds. Incorporating additional endpoints in this type of study would undoubtedly enrich our findings. The ecotoxicological effects of glyphosate on aquatic macrophytes, such as *L. minor*, include, among other factors, the dilution of this substance in water and the application of high doses of glyphosate on land near reservoirs—topics well discussed in the review by Villamar-Ayala et al. (2019). However, none of these studies consider the impact of substrates with varying organic content on glyphosate and AMPA toxicity to *L. minor*, nor do they address how this toxicity diminishes over time, as shown in the present work.

In this research, the Microtox biotest revealed higher herbicide toxicity to the organism in the substrate with the lowest organic matter content after 1 day compared to longer exposure times. This clearly indicates that the organic matter content of the medium is an important factor when assessing glyphosate toxicity. The validity of our use of the Microtox biotest, which measures the inhibition of bioluminescence in *V. fischeri* bacteria to evaluate glyphosate toxicity, is supported by the work of other researchers. For example, Bonnet et al. (2007) and Sihtmäe et al. (2013) successfully employed *V. fischeri* in their studies on glyphosate toxicity. To illustrate, Sihtmäe et al. (2013) found that the toxicity of Roundup formulations and the isopropylamine (IPA) salt of glyphosate to *V. fischeri* was in the range of 5.4–7.6 mg AE/L (acid equivalents per liter) at 30 min EC_50_. Additionally, Roundup Quick™ was slightly more toxic than the IPA salt of glyphosate and Roundup Max™. Sihtmäe et al. (2013) demonstrated that changes in the bacterial bioluminescence of *V. fischeri* are a very rapid and sensitive endpoint for assessing glyphosate toxicity. For Roundup Quick™, a strong effect on bacterial luminescence was observed within the first few seconds, indicating that glyphosate quickly damages the bacterial cell membrane. In contrast, the effects of Roundup Max™ and IPA salt of glyphosate were not as rapid; however, the final EC_50_ values after 30 min of exposure were comparable to those of Roundup Quick™ (Sihtmäe et al. 2013). It is important to note that the Microtox test uses marine bacteria *V. fischeri*, and the reference media tested without glyphosate also exhibited a certain baseline level of toxicity for these organisms. Therefore, in our study, the observed effects of glyphosate toxicity may have been less pronounced for some variants—both due to differences in substrate organic matter content and the time elapsed since herbicide application. In future experiments, instead of using soil extracts with varying organic matter content, we could analyze aqueous solutions with different nutrient levels and add glyphosate and AMPA to them.

Finally, it is worth noting that the toxicity of herbicides containing glyphosate is influenced not only by the factors mentioned earlier but also by their formulation and the addition of adjuvants (e.g., Duke 2020; Padilla and Selim 2020; Singh et al. 2020a, b). The complexity of the factors affecting glyphosate and AMPA toxicity clearly demonstrates that using standardized ecotoxicological methods, such as biotests, combined with chemical analysis of substrates, enables a comprehensive assessment of the actual impact—not only of glyphosate/AMPA but also of other substances—on the environment.

To summarize the results of the ecotoxicological studies, the number of endpoints should be increased in future research, as both glyphosate and AMPA affect many vital processes in plants and microorganisms. It would also be valuable to conduct simultaneous analyses of glyphosate and AMPA to assess their toxicity to organisms independently. The use of high herbicide doses in this study was important, since most studies employ much lower doses. Additionally, it is worthwhile to continue using substrates with varying organic matter content, as these influence the rate of glyphosate and AMPA degradation, and to extend the study duration to a longer time scale (up to two years). It is also advisable to include a greater variety of test species. Furthermore, in bioassays using *L. minor* and V*. fischeri* instead of soil extracts, efforts could be made to analyze the effects of glyphosate and AMPA in waters with different nutrient levels. Although biotest studies do not replicate all environmental conditions, they undoubtedly provide valuable insights, help break down complex environmental parameters into their constituent factors, and facilitate the replication of experimental conditions across different laboratories.

### The amount of nitrogen and phosphorus in substrates with varying organic matter content

Our study also allowed us to examine whether, following the disappearance of glyphosate and AMPA toxicity, nutrient enrichment of the tested media might occur, leading to better growth of organisms compared to the control. The most effective tool for this research was the Phytotoxkit bioassay using *L. sativum*, combined with chemical analysis results. The roots of *L. sativum* plants grew better in agricultural soil with the herbicide added than in the control substrate, even during the early stages of plant growth—starting from the sixth month after herbicide application. However, after 24 months, a significant stimulation of *L. sativum* root growth was observed in the agricultural soil treated with Roundup compared to the control. Chemical analysis of nitrogen and phosphorus content revealed that, in the samples containing only agricultural soil or soil mixed with sand, the levels of nitrogen and phosphorus (including available forms) were higher in the herbicide-treated samples at all time points than in the corresponding controls. This trend was not always observed in the pure sand samples, which can be explained by the slower decomposition of glyphosate and AMPA in this substrate. It remains an open question whether the growth stimulation observed in *L. sativum* following the disappearance of glyphosate and AMPA toxicity was due to increased nitrogen and phosphorus levels or to a hormetic effect caused by very low doses of these substances in agricultural soil and soil mixed with sand. Low doses of glyphosate have been shown to induce hormesis, stimulating plant growth and yield under certain conditions. For example, in white oat, glyphosate hormesis was more pronounced under low N availability, leading to increased dry weight and grain yield. Similarly, low doses of glyphosate enhanced growth parameters in coffee seedlings and marandu grass, with effects such as increased stem diameter, leaf area, and phosphorus content (Silva et al. 2020; Codognoto et al. 2021; Terra et al. 2021).

The results obtained may have been influenced by changes in the microbial composition of the studied substrates, a factor that was not examined in this study. Insights from other researchers suggest that the use of herbicides on railway embankments negatively impacts microbial activity, which may extend the decomposition time of the herbicides in such poor media (Cederlund and Stenström 2004). Conversely, the lack of growth stimulation in the tested organisms after shorter periods following herbicide application can be explained by the continued presence of glyphosate and AMPA in the substrates, exerting toxic effects on the organisms. As Cederlund and Stenström (2004) point out, one of the objectives of applying herbicides to railway tracks—beyond weed control—is to reduce organic matter accumulation, as it binds water and can destabilize the embankment. Consequently, railway embankments tend to have low organic matter content, leading to reduced biomass and microbial activity. They also note that herbicides such as glyphosate, which degrade readily in fertile agricultural soils, can persist and leach in substrates with low organic matter content, such as those found in railway embankments (Cederlund and Stenström 2004).

Phosphatases play an important role in soil by facilitating the conversion of organic phosphorus compounds into inorganic phosphates. The primary sources of phosphatases in the soil environment are soil microorganisms. Literature data indicate that the application of glyphosate, for example, can alter the activity of enzymes involved in the metabolism of phosphorus compounds in loamy sand. The observed effects depend on various factors, including the type of formulation used, the herbicide dose, and the incubation temperature (Płatkowski and Telesiński 2015a, b, 2016a, b). In the cited studies, among the enzymes tested, inorganic pyrophosphatase was the most sensitive to glyphosate presence in loamy sand. The observed inhibition of the enzyme's average activity under glyphosate exposure at all tested doses occurred at incubation temperatures of 4 °C and 30 °C. Conversely, the introduction of glyphosate as an ammonium salt altered the available phosphorus content and affected the activity of enzymes involved in the transformation of this element in loamy sand. Following the application of Roundup 360 SL, which contains glyphosate isopropylamine salt and polyethoxylated tallow amine, a statistically significant positive correlation was observed between inorganic pyrophosphatase activity and the soil’s available phosphorus content (Płatkowski and Telesiński 2015a, b, 2016a, b). Płatkowski and Telesiński (2017) also showed that the application of preparations containing glyphosate in the form of isopropylamine salt (Roundup 360 SL) and potassium salt (Roundup 360 Plus) to sandy loamy clay causes significant changes in soil phosphatase activities. Among the phosphatases tested, inorganic pyrophosphatase was the most sensitive to the presence of glyphosate-containing preparations in the soil. They also found that the type of glyphosate formulation had the greatest effect on phosphotriesterase activity, while the activity of the remaining soil phosphatases was mainly influenced by the duration of the experiment. These observations confirm the results of our research, suggesting the possibility of an increase in phosphorus content in the substrate following the decomposition of glyphosate and AMPA. However, it's important to note that we used Roundup Ultra 170 SL, tested substrates with varying organic matter content, and that our experimental design and objectives were different. Our research is just the first step. Confirming the hypothesis that glyphosate, once degraded, may increase nitrogen and phosphorus levels in the soil—especially in soils with low organic matter—requires further investigation, including a comprehensive analysis of samples from areas where high doses of this herbicide are applied.

It is also important to remember that some microorganisms can use glyphosate as a source of phosphorus and/or nitrogen (Zhan et al. 2018). Therefore, the potential enrichment of the substrate with these nutrients after glyphosate decomposition may have some environmental impact. Although this hypothesis clearly requires further investigation, existing literature suggests that there could be an effect on the environment. For example, studies have shown that glyphosate can stimulate the growth of mycorrhizal fungi (Laatikainen and Heinonen-Tanski 2002). Additionally, it was found that the presence of glyphosate in the soil could lead to an increase in microbial populations and their activity, as observed in Brazil where the herbicide was used (Araújo et al. 2003). Furthermore, Carranza et al. (2017) conducted studies on the effects of herbicides on soil microflora and their degradation processes. They demonstrated that fungi from the *Aspergillus* genus (*Aspergillus flavus* and *Aspergillus niger*) are the primary microorganisms responsible for degrading glyphosate. All strains showed increased growth (ranging from 24 to 44%) on a medium with glyphosate as the sole source of nitrogen or phosphorus compared to the control (Carranza et al. 2017). Additionally, *Penicillium chrysogenum* was isolated from a medium where glyphosate was the only source of nitrogen. The presence of the herbicide also stimulated the growth of *Penicillium chrysogenum* (Klimek et al. 2001). However, as mentioned earlier when discussing our research, this may be a hormetic effect of low doses of glyphosate/AMPA.

Sun et al. (2019) studied the degradation of glyphosate and its metabolites and successfully used phosphate oxygen isotopes to confirm the biological availability of glyphosate-derived phosphorus in a simulated soil–water system. They found that AMPA was 3–6 times more resistant to degradation than glyphosate. The distribution of glyphosate-derived phosphorus in soil was also investigated by Sun et al. (2019). Approximately half of the glyphosate-derived phosphorus transferred into the readily bioavailable phosphorus pool. They also discovered that microorganisms were more efficient at utilizing and recycling glyphosate-derived phosphorus (Sun et al. 2019). Authors indicate that phosphorus loads resulting from extensive glyphosate application cannot be overlooked, as they can serve as a source of phosphorus not only for microorganisms but also for plants. In turn, conditionally unavailable phosphorus pools may be further transported into open water systems through leaching or soil erosion, thereby increasing the risk of water pollution (Sun et al. 2019). Our study demonstrated that the growth of *L. sativum* was stimulated in soil treated with Roundup once the herbicide’s toxicity had dissipated after 24 months—an encouraging finding compared to the studies cited. These results suggest the possibility that soil may become enriched with nutrients (N and P) following the application of the herbicide Roundup. However, this topic still requires further research. A better understanding of the behavior of glyphosate and AMPA in the soil is important for improving environmental protection and adjusting herbicide use legislation accordingly. It is also worth noting that the enrichment of substrates with low organic matter with nitrogen and phosphorus after glyphosate application may partly explain, for example, the occurrence of *G. robertianum* in railway areas, even though this species prefers more fertile forest habitats (Bemowska-Kałabun et al. 2021). However, this hypothesis requires further research.

## Conclusion

This study provides initial insights into the persistence and ecological impact of glyphosate and its primary metabolite, AMPA, across different substrate types. Our findings suggest that the toxicity of these compounds can last from several months to two years, depending on, among others, the substrate’s organic matter content (and other parameters, as shown by literature data, that were not tested here). Specifically, in sand-only environments with low organic matter, both glyphosate and AMPA remained detectable and potentially toxic for over 12 and 24 months, respectively. Substrates with high organic matter appeared to facilitate more rapid glyphosate and AMPA degradation, with toxic effects diminishing within approximately three months in this type of substrate. Moreover, the observed increases in phosphorus and nitrogen levels following herbicide application highlight potential nutrient alterations that could influence ecosystem processes. However, this requires further research. While these results are based on controlled laboratory experiments, they underscore the complexity of glyphosate’s environmental fate and effects. Notably, the study emphasizes the importance of including both glyphosate and AMPA in ecotoxicological assessments, as the latter’s persistence and toxicity are often overlooked despite its ecological relevance.

These findings serve as a preliminary step towards understanding the long-term ecological risks associated with glyphosate use. They also highlight the need for further research—particularly field studies—to evaluate the real-world implications of repeated and high-dose applications of herbicide, especially in environments with low organic matter where degradation may be slower. Future investigations should aim to clarify the potential for the development of glyphosate tolerance in local flora and the broader impacts on ecosystem health. It is also worth investigating whether, under field conditions, there is indeed an increase in nitrogen and phosphorus levels following the application of high doses of glyphosate—particularly in environments low in organic matter—and examining the potential impact on the natural environment. Overall, a comprehensive approach combining chemical analysis with ecotoxicological testing is essential for informed regulation and sustainable management of herbicide use.

## Supplementary Information

Below is the link to the electronic supplementary material.

ESM1 (XLSX 58.8 KB)

## Data Availability

Data will be available on reasonable request and in the repositories of the University of Warsaw.
